# A Phosphoproteomics Study of the Soybean *root necrosis 1* Mutant Revealed Type II Metacaspases Involved in Cell Death Pathway

**DOI:** 10.3389/fpls.2022.882561

**Published:** 2022-07-19

**Authors:** Feifei Wang, Priyanka Das, Narinder Pal, Ruchika Bhawal, Sheng Zhang, Madan K. Bhattacharyya

**Affiliations:** ^1^Department of Agronomy, Iowa State University, Ames, IA, United States; ^2^Proteomics and Metabolomics Facility, Institute of Biotechnology, Cornell University, Ithaca, NY, United States

**Keywords:** proteomics, phosphoproteomics, *root necrosis*, metacaspases, cell death, soybean

## Abstract

The soybean *root necrosis 1* (*rn1*) mutation causes progressive browning of the roots soon after germination and provides increased tolerance to the soil-borne oomycete pathogen *Phytophthora sojae* in soybean. Toward understanding the molecular basis of the *rn1* mutant phenotypes, we conducted tandem mass tag (TMT)-labeling proteomics and phosphoproteomics analyses of the root tissues of the *rn1* mutant and progenitor T322 line to identify potential proteins involved in manifestation of the mutant phenotype. We identified 3,160 proteins. When the *p*-value was set at ≤0.05 and the fold change of protein accumulation between *rn1* and T322 at ≥1.5 or ≤0.67, we detected 118 proteins that showed increased levels and 32 proteins decreased levels in *rn1* as compared to that in T322. The differentially accumulated proteins (DAPs) are involved in several pathways including cellular processes for processing environmental and genetic information, metabolism and organismal systems. Five pathogenesis-related proteins were accumulated to higher levels in the mutant as compared to that in T322. Several of the DAPs are involved in hormone signaling, redox reaction, signal transduction, and cell wall modification processes activated in plant–pathogen interactions. The phosphoproteomics analysis identified 22 phosphopeptides, the levels of phosphorylation of which were significantly different between *rn1* and T322 lines. The phosphorylation levels of two type II metacaspases were reduced in *rn1* as compared to T322. Type II metacaspase has been shown to be a negative regulator of hypersensitive cell death. In absence of the functional Rn1 protein, two type II metacaspases exhibited reduced phosphorylation levels and failed to show negative regulatory cell death function in the soybean *rn1* mutant. We hypothesize that Rn1 directly or indirectly phosphorylates type II metacaspases to negatively regulate the cell death process in soybean roots.

## Introduction

Soybean [*Glycine max*, (L.) Merr.] is one of the most important legumes worldwide and is an important source of both vegetable proteins and oil for human nutrition. However, soybean production is often impeded by attacks from a large number of pathogens including oomycetes, nematodes, fungi, bacteria, and viruses. Soil-borne root pathogens can cause root necrosis and rot resulting in significant yield reductions (Liu et al., 2016; Strom et al., 2020). Plants have evolved with defense mechanisms to protect against pathogen attacks. One of the defense mechanisms is the hypersensitive response (HR), also known as programmed cell death (PCD), which is characterized by rapid death of plants cells that are in contact with the invading pathogens. This is one of the most common and effective host defense responses (Muthamilarasan and Prasad, 2013; Balint-Kurti, 2019). PCD is a ubiquitous and genetically regulated process consisted of activation of finely controlled signaling pathways leading to cellular suicide (Pontier et al., 1998; Salguero-Linares and Coll, 2019; Noman et al., 2020).

Lesion mimic mutants (LMMs), characterized by sudden spontaneous cell death, have become powerful resources in studying the role of cell death in defense response and molecular mechanisms regulating PCD and the HR in plants. Different recessive lesion mimic mutations can lead to PCD with or without activation of host defense responses (Jones and Dangl, 1996; Mittler and Lam, 1996; Lorrain et al., 2003; Bruggeman et al., 2015). LMMs have been reported in many plant species including *Arabidopsis* (Zhang et al., 2008), barley (Jorgensen, 1992; Rostoks et al., 2006), birch (Li et al., 2017), maize (Walbot, 1991; Gray et al., 1997), rice (Mizobuchi et al., 2002; Jiao et al., 2018; Zhang et al., 2019), soybean (Kosslak et al., 1996), and wheat (Kong et al., 2020). A number of genes regulating the development of LMM phenotypes have been mapped and cloned. Several rice *LMM* genes have been cloned and the major pathways regulated by these genes have been identified. These include ROS pathways (Takahashi et al., 1999; Wu et al., 2008), chlorophyll synthesis (Nishimura et al., 2019; Cui et al., 2020), fatty acid and lipid biosynthesis (Gao et al., 2019, 2020), signal transduction (Fekih et al., 2015), and kinase signaling pathways (Zhou et al., 2016; Zhang et al., 2019).

In soybean *Glycine max lesion mimic mutant 2-1* (*Gmlmm2-1*), which displays a light-dependent cell death phenotype, is controlled by the *GmLMM2* gene encoding a coproporphyrinogen III oxidase involved in tetrapyrrole biosynthesis (Ma et al., 2020). Investigation of the spotted leaf-1 (*spl-1*) mutant, another LLM in soybean, led to identification of a candidate *Glyma.04g242300* gene that showed high similarity to the *Arabidopsis At2G02850* gene encoding a plantacyanin, a member of the plant-specific phytocyanin sub-family of blue copper proteins involved in the electron transport chain of photosynthesis (Al Amin et al., 2019). A forward genetic screen for autoimmunity-related LMMs in soybean detected two allelic mutants, which carry mutations in *Glyma.13G054400* encoding a malectin-like receptor kinase (Wang et al., 2020a). A soybean LMM, NT302 showing chlorotic and spontaneous lesions on leaves at the R3 pod-stage is governed by lack of a functional *GmHPL* gene encoding a hydroperoxide lyase. *GmHPL* is significantly induced in response to methyl jasmonate treatment, wounding, and infestation with common cutworm (Wang et al., 2020b). All these LMMs were identified based on spontaneous necrosis in leaves.

**FIGURE 1:**
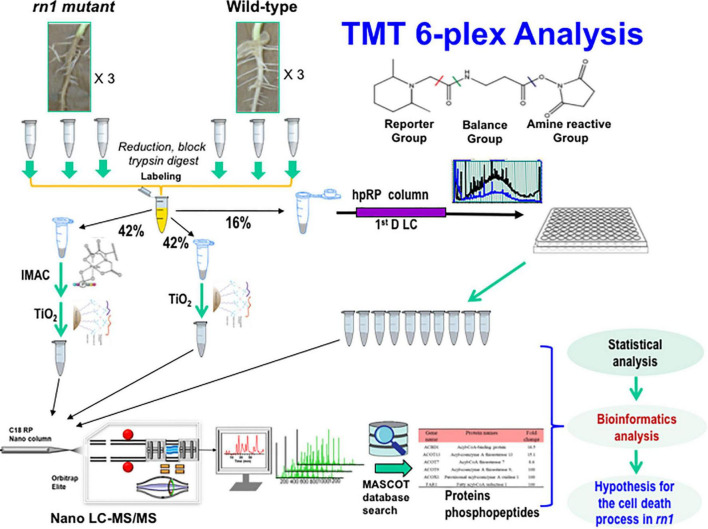
Experimental design and schematic diagram of the workflow for quantitative proteomics of the soybean *rn1* mutant and T322 lines. The T322 is the wild-type progenitor of the *rn1* mutant line. Soybean root samples of the *rn1* mutant and T322 lines in triplicate were analyzed in parallel by TMT 6-plex based quantitative proteomics and phosphoproteomics analyses. TMT labeled peptides were divided to three aliquots and used by high pH fractionation for global proteomics, TiO_2_ enrichment and IMAC-TiO_2_ sequential enrichment for phosphoproteomics, respectively. About 16% of total labeled peptides were used for global proteomics and 84% used for phosphoproteomics study. The 10 high-pH RPLC fractions and 2 enriched samples were subjected to nanoLC-MS/MS analysis and subsequent database search using Mascot. After statistical analyses of the global proteomics data and quantitative phosphopeptides, the differentially accumulated proteins, and phosphoproteins with significantly changed phosphorylated peptides/sites were conducted to identify differentially regulated proteins and phosphoproteins by the Rn1 protein for bioinformatics analysis.

Thirty root necrotic mutants induced mostly by an endogenous transposon, treatment with the chemical mutagen ethyl methanesulfonate or spontaneously have been reported to be allelic suggesting that most likely a single gene regulates the cell death pathway in soybean roots (Palmer et al., 2008). The roots of homozygous *root necrosis 1* (*rn1*) plants, even under axenic conditions, turn brown soon after germination and exhibit increased tolerance to *Phytophthora sojae*, the root-borne oomycete pathogen that causes root and stem rot disease in soybean (Kosslak et al., 1996). The lack of a functional *Rn1* gene causes a progressive browning in the roots between 3 and 5 days after germination and is associated with the accumulation of phytoalexins and pathogenesis-related proteins (Kosslak et al., 1997; Figure 1 and Supplementary Figure 1). The browning is caused by the oxidation of phenolic compounds and is a characteristic symptom of the HR associated with wounding or pathogen attack (Lamb, 1994). Improvement in tolerance to *P. sojae* is considered to be associated with the onset of defense gene activation in *rn1* mutants. The *Rn1* gene is yet to be cloned. It is also unknown which genes, proteins or pathways are regulated by *Rn1*. For better understanding molecular basis of the root necrotic mutant 1 (*rn1*) phenotype in soybean, we applied proteomics and phosphoproteomics approaches that have been extensively used in recent years for exploring the molecular basis of plant growth and responses to biotic and abiotic stresses in plant species including *Arabidopsis* (Umezawa et al., 2013), *Brachypodium distachyon* (Lv et al., 2014; Yuan et al., 2016), rice (Hou et al., 2015; Fang et al., 2019), wheat (Hu et al., 2015), maize (Cao et al., 2019; Zhao et al., 2019), and barley (Ishikawa et al., 2019a,b). We have applied tandem mass tags (TMT)-based quantitative liquid chromatography-tandem mass spectrometry (LC-MS/MS) proteomics and phosphoproteomics approaches to compare the proteomes and phosphoproteomes of the wild-type 322 and *rn1* mutant root tissues and identified 22 phosphoproteins including two highly identical type II metacaspases with significantly different phosphorylation levels between the *rn1* mutant and the “wild-type T322” progenitor lines.

## Materials and Methods

### Plant Material and Growing Conditions

The seeds of the progenitor soybean cultivar T322 and *rn1* mutant (T328H) were planted on germinating papers and incubated under the following growing conditions: 24–26°C day/19–21°C night, 16 h light/8 h dark, and 350 μmol photons m^–2^ s^–1^ light intensity. Germinating seedlings were phenotyped starting 5 days following sowing for visible necrotic phenotypes. The comparable amounts of necrotic root tissues, as soon as necrotic symptoms started to appear, from the *rn1* mutant and healthy tissues from the wild-type T322 plants were harvested and frozen in liquid N_2_. The root tissues of random plants were pooled to obtain three replications for each of the wild-type T322 and *rn1* mutant plants for proteomics and phosphoproteomics studies.

### Preparation of Protein Samples

The proteins from roots of soybean were extracted following the method of Toorchi et al. (2009). Phenol extraction buffer, a mixture of equal volume of extraction buffer and buffered phenol, was prepared just before use. The extraction buffer contains 100 mM Tris–HCl, pH 8.8, 10 mM w/v EDTA, 900 mM w/v sucrose, and 0.4% v/v 2-mercaptoethanol. Buffered phenol was prepared by dissolving phenol in Tris buffer and adjusting the pH to 8.8 with HCl. About 5 *g* tissue sample was ground to powder under liquid N_2_ using a pestle and mortar. Approximately 10 ml phenol extraction buffer was added to the powdered 5 *g* tissue in a 50 ml tube and protein was extracted by agitating for 30 min at 4°C. The content was centrifuged at 2,800 *g* for 30 min at 4°C. The upper phase was then transferred to a fresh 50 ml tube. Protease and phosphatase inhibitors were added to 1% concentration. Ice-cold ammonium acetate/methanol solution (100 mM w/v ammonium acetate in 100% methanol) was added to the upper phase in a ratio of 5:1, mixed and incubated at -20°C to precipitate the phenol extracted proteins. The precipitate was collected after centrifugation at 2,800 *g* for 30 min at 4°C. The pellet was washed twice with of ice-cold ammonium acetate/methanol solution, then twice with ice-cold 80% acetone solution, and finally with ice-cold 70% ethanol. The pellet was solubilized and denatured in a final concentration of 100 mM phosphate buffer pH 7.8, containing 6M urea and 2% CHAPS, 0.2% Triton X-100, 0.1% SDS and 10 mM DTT, sonicated for 5 min and votexed for 0.5 h until completely solubilized. The protein concentration for each sample was determined by Bradford assay, and further quantified by running on a precast NOVEX 12% Tris/Glycine mini-gel (Invitrogen, Carlsbad, CA, United States) along with a series of amounts of *E. coli* lysates (2, 5, 10, and 20 μg/lane). The SDS gel was visualized with colloidal Coomassie blue stain (Invitrogen), imaged by Typhoon 9400 scanner followed by ImageQuant TL 8.1 (GE Healthcare).

### Tandem Mass Tags Labeling and Phosphopeptide Enrichment and Detection

The proteomics and phosphoproteomics analyses were conducted at the Proteomics and Metabolomics Facility, Cornell University to identify the differentially accumulated proteins (DAPs) and phosphopeptides/sites. For TMT labeling, a total of 200 μg protein of each sample was reduced with 9.5 mM tris(2-carboxyethyl)phosphine for 1 h at room temperature, alkylated with 17 mM iodoacetamide for 1 h in the dark and then quenched by additional of 20 mM Dithiothreitol (DTT). The alkylated proteins were precipitated by adding 6 volumes of ice-cold acetone and incubating at -20°C overnight, and reconstituted in 90 μL of 100 mM triethyl-ammonium bicarbonate. Each sample was digested with 18 μg trypsin for 18 h at 37°C. The TMT 6-plex labels (dried powder) were reconstituted with 45 μL of anhydrous ACN prior to labeling and added with 1: 2 ratio to each of the tryptic digest samples for labeling over 1 h at room temperature. The peptides from the following six samples (mut1, mut2, mut3, WT1, WT2, and WT3) were mixed each TMT tag with 126-tag, 127-tag, 128-tag, 129-tag, 130-tag, and 131-tag, respectively. After checking label incorporation using Orbitrap Elite (Thermo-Fisher Scientific, San Jose, CA, United States) by mixing 1 μL aliquots from each sample and desalting with strong cation-exchange (SCX) ziptip (Millipore, Billerica, MA, United States), the six samples in each set were pooled, evaporated to dryness and subjected to a PolyLC SCX cartridge (PolyLC Inc. Columbia, MD, United States) and Sep-Pak C18 cleanup (Waters, Milford, MA, United States) according to the manufacturer’s instructions. The clean samples were then divided to three aliquots: one aliquot with 200 μg peptides used for subsequent high-pH reverse-phase fractionation into 10 fractions as described previously (Yang et al., 2011). The rest two aliquots (500 μg peptides each) were used for enrichment of phosphopeptides.

Enrichment of phosphopeptides by TiO_2_ beads: TiO_2_ enrichment was conducted using a TiO_2_ Mag Sepharose kit (from GE Healthcare) for each 500 μg aliquot. The TMT 6-plex tagged tryptic peptides were reconstituted in 400 μL of binding buffer (1M glycolic acid in 80% acetonitrile, 5% TFA). The TiO_2_ slurry (75 μL) was used and incubated with the sample for 30 min at 1,800 rpm vortex. After washing the beads with washing buffer (80% acetonitrile, 1%TFA), the phosphopeptides were eluted with 100 μL of elution buffer (5% ammonium hydroxide) twice. The eluted fraction was dried and reconstituted in 25 μL of 0.5% formic acid for subsequent nano scale LC-MS/MS analysis. The second aliquot (500 μg peptides) was used for sequential enrichment of phosphopeptides by IMAC and TiO_2_. The IMAC enrichment was carried out using a PHOS-Select Iron Affinity Gel (Sigma P9740) kit following the vendor-recommended procedure. The sequential enrichment steps were conducted as previously reported (Gao et al., 2017).

### Nano Liquid Chromatography-Tandem Mass Spectrometry Analysis

The nanoLC-MS/MS analysis was carried out using an Orbitrap Elite (Thermo-Fisher Scientific, San Jose, CA, United States) mass spectrometer equipped with nano ion source using high energy collision dissociation (HCD). The Orbitrap is equipped with a “CorConneX” nano ion source (CorSolutions LLC, Ithaca, NY, United States) coupled with the UltiMate3000 RSLCnano (Thermo, Sunnyvale, CA, United States). The nanoLC-MS/MS was operated in top-15 data-dependent acquisition HCD-MS/MS mode with multiple charged ions above a threshold ion count of 8,000 with normalized collision energy of 37% as described previously for 10 fractions of global proteome (Wang et al., 2014) and two enriched fractions of phosphoproteomics (Gao et al., 2017).

### Protein Identification and Quantification

MS raw data files were converted into MGF files for identification and relative quantitation using Proteome Discoverer version (PD) 1.4 (Thermo-Fisher Scientific). A subsequent database search was performed with Mascot Daemon version 2.3 (Matrix Science, Boston, MA, United States) against the Soybean protein RefSeq database (88,647 entries) downloaded on Dec. 1st, 2014, from NCBInr. The default Mascot search settings were as follows: MS peptide tolerance, 15 ppm; fragment mass tolerance, 0.1 Da; trypsin digestion allowing two missed cleavage with fixed modifications of cysteine carbamidomethylation, N-terminal TMT6plex, and lysine TMT6plex; and with deamidation (NQ), oxidation (M), and phosphorylation (STY) as variable modifications. To estimate the false discovery rate (FDR) for a measure of identification certainty in database search, we employed the target–decoy strategy of Elias and Gygi (2007). Specifically, an automatic decoy database search was performed in Mascot by choosing the decoy checkbox in which a random sequence of database is generated and tested for raw spectra as well as the real database. To reduce the probability of false peptide identifications, we considered as identified only those peptides with significant scores (≥25) at the 99% confidence interval by a Mascot probability analysis greater than “identity.” This required that each confident protein identification involve at least two unique peptide identifications indicated in Mascot. In addition, to confidently locate phosphorylation sites, the phosphoRS 3.0 node integrated in PD 1.4 workflow was also used to cross-validate the results of Mascot as reported (Gao et al., 2017).

For protein quantification, we used those proteins that were identified in all six TMT channels with at least two unique peptides in a TMT experiment (Krey et al., 2018). Intensities of the reporter ions from TMT 6-tags upon fragmentation were used for quantification, and the relative quantitation ratios were normalized to median protein ratio for the 6-plex in the dataset. We used Mascot database search engine for processing TMT-based quantitative global proteomics and phosphoproteomics datasets, in which we employed the target–decoy strategy as a measure of identification certainty in database search with 1% FDR as a cutoff. Quantitative comparison of the triplicate samples between the two groups was undertaken with Microsoft Excel software using a student *t*-test. To identify DAPs related to root-necrosis caused by the *rn1* mutation, the quantifiable proteins were tabulated and their differential abundance ratio (treated/control) was log_2_ transformed. The log_2_ fold values, hereafter referred as fold change (FC), were fit to a normal distribution to obtain the standard deviation of the quantified proteome. The DAPs between *rn1* mutant and wild-type 322 were listed using a cutoff of FC value between the *rn1* and wild-type 322 based on the criteria of the *p*-values ≤ 0.05 and FC values ≥ 1.5 or ≤0.67.

The mass spectrometry proteomics data have been deposited to the ProteomeXchange Consortium *via* the PRIDE partner repository with the dataset identifier PXD032226.

### Bioinformatics Analysis of Proteins

The transcripts of genes encoded the DAPs and phosphorylated proteins were obtained using the fragments per kilobase of exon model per million mapped (FPKM) reads according to the file (genes.fpkm_tracking) listed in the directory of *Glycine max* Wm82.a2.v1^1^ of the Phytozome V13 database (Severin et al., 2010; Goodstein et al., 2012). A stringent filtering criterion of FPKM value 1.0 (equivalent to one transcript per cell; Mortazavi et al., 2008) in the root was used to identify the root-specific expression level of the genes encoding DAPs and phosphoproteins.

The DAPs and phosphorylated proteins were classified into different categories of gene ontology (GOs), biological processes and molecular functions by using GOatools^2^.

Molecular functions of the identified DAPs were annotated by Plant MetGenMap Mercator (Joung et al., 2009) and Kyoto Encyclopedia of Genes and Genome (KEGG) databases (Kanehisa et al., 2017), which were separately performed online^3^. The pathways of the DAPs including 12 phosphorylated DAPs were generated using MapMan software^4^.

## Results

### Identification of Differentially Accumulated Root Necrosis-Related Proteins Regulated by *Rn1*

The *rn1* mutant exhibited a root necrotic phenotype and enhanced tolerance to the oomycete pathogen *P. sojae* (Kosslak et al., 1997). To better understand the mechanisms regulated by the Rn1 protein, we compared the root proteomes of the wild-type T322 (*Rn1*) and mutant T328H (*rn1*) lines. We used a TMT mass spectrometry-based quantitative proteomics approach to identify the proteins that were differentially accumulated between the lines. Six TMTs were used to label three replicates of necrotic root tissues of the *rn1* (T328H) mutant and three of healthy root tissues from the wild-type progenitor T322 plants (Figure 1 and Supplementary Figure 1).

We detected 3,160 proteins, of which 2,180 were found in at least two replications of both necrotic and healthy root tissues. Of the 2,180 proteins, the 150 were DAPs between *rn1* and T322 roots, regulated presumably by Rn1, which were selected for further investigation (Table 1). Among the 150 DAPs, 118 showed ≥ 1.5-fold increase and 32 showed ≤ 0.67-fold decrease in the necrotic root tissues as compared to that in the healthy root tissues at *p* ≤ 0.05. Genes encoding 138 of the 150 DAPs showed detectable expression levels (Transcript Number ≥ 1) in roots of the soybean cultivar Williams 82 (Table 1).

**TABLE 1:** The 150 differentially accumulated proteins between *rn1* and T322 wild-type lines.

Protein	Annotation	^1^Fold change	*p*-value	^2^Transcript
**The 118 proteins with enhanced accumulation in the *rn1* mutant as compared to that in the wild-type T322 line**				
Glyma.13G251600	CAP (Cysteine-rich secretory proteins, Antigen 5, and Pathogenesis-related 1 protein)	25.56 ± 16.29	0.01	2.64
Glyma.07G243500	MLP-like protein 423	8.08 ± 3.27	0.00	675.96
Glyma.17G030400	MLP-like protein 423	7.29 ± 2.95	0.00	312.78
Glyma.03G206400	Eukaryotic translation initiation factor 3C	4.97 ± 1.36	0.00	30.48
Glyma.08G308100	RNI-like superfamily protein	4.96 ± 3.23	0.01	0.05
Glyma.17G207900	K + transporter 1	4.57 ± 3.90	0.04	0.03
Glyma.08G341500	Kunitz trypsin inhibitor 1	4.26 ± 2.59	0.01	0.90
Glyma.11G062600	Cytochrome P450, family 71, subfamily B, polypeptide 34	4.07 ± 1.14	0.00	76.14
Glyma.10G060800	Pathogenesis-related thaumatin superfamily protein	3.98 ± 2.41	0.01	17.24
Glyma.10G246300	Cupin family protein	3.52 ± 2.50	0.03	0.51
Glyma.09G158500	Unknown	3.44 ± 1.88	0.01	0.67
Glyma.20G169200	Peroxidase superfamily protein	3.38 ± 1.35	0.00	109.71
Glyma.03G147700	Disease resistance-responsive (dirigent-like protein) family protein	3.34 ± 1.46	0.00	94.94
Glyma.20G116100	Ribosomal protein L15	3.30 ± 2.43	0.04	19.42
Glyma.18G177000	Elicitor-activated gene 3-2	3.25 ± 1.14	0.00	6.08
Glyma.12G141900	Flavodoxin-like quinone reductase 1	3.23 ± 0.85	0.00	21.80
Glyma.01G187700	S-adenosyl-L-methionine-dependent methyltransferases superfamily protein	3.23 ± 1.28	0.00	705.31
Glyma.15G211500	Kunitz family trypsin and protease inhibitor protein	3.14 ± 1.38	0.00	122.72
Glyma.06G260200	NAD(P)-linked oxidoreductase superfamily protein	3.14 ± 0.67	0.00	11.73
Glyma.19G245400	Pathogenesis-related 4	3.13 ± 1.12	0.00	77.07
Glyma.03G040400	Lipid transfer protein 1	3.04 ± 2.03	0.03	425.90
Glyma.07G139700	Glutathione S-transferase TAU 8	2.88 ± 0.73	0.00	182.31
Glyma.08G175200	Glutathione S-transferase TAU 19	2.83 ± 0.61	0.00	209.69
Glyma.17G164200	Late embryogenesis abundant protein, group 6	2.78 ± 1.11	0.00	13.44
Glyma.20G205800	Serine protease inhibitor, potato inhibitor I-type family protein	2.71 ± 0.83	0.00	1327.82
Glyma.08G341400	Kunitz family trypsin and protease inhibitor protein	2.71 ± 0.64	0.00	746.05
Glyma.13G223700	Protein of unknown function (DUF1264)	2.68 ± 1.12	0.01	1.04
Glyma.01G219400	Glutathione peroxidase 6	2.67 ± 0.64	0.00	61.10
Glyma.17G040800	Late embryogenesis abundant domain-containing protein/LEA domain-containing protein	2.65 ± 0.53	0.00	1.98
Glyma.08G341300	Kunitz family trypsin and protease inhibitor protein	2.63 ± 0.88	0.00	90.42
Glyma.02G042500	Basic chitinase	2.59 ± 1.37	0.02	173.64
Glyma.05G198000	Low-molecular-weight cysteine-rich 69	2.57 ± 1.45	0.03	2.09
Glyma.01G123100	PEBP (phosphatidylethanolamine-binding protein) family protein	2.53 ± 0.69	0.00	27.56
Glyma.16G208900	Unknown	2.51 ± 0.99	0.01	0.15
Glyma.13G237700	Late embryogenesis abundant protein (LEA) family protein	2.49 ± 0.73	0.00	1.97
Glyma.03G056000	Stress induced protein	2.49 ± 0.91	0.00	12.77
Glyma.15G026300	Lipoxygenase 1	2.49 ± 1.07	0.01	2.37
Glyma.08G088000	1-cysteine peroxiredoxin 1	2.49 ± 0.60	0.00	0.64
Glyma.17G030100	MLP-like protein 423	2.48 ± 0.75	0.00	1608.94
Glyma.04G054400	17.6 kda class II heat shock protein	2.46 ± 0.79	0.00	5.49
Glyma.03G052200	PEBP (phosphatidylethanolamine-binding protein) family protein	2.42 ± 0.62	0.00	2.33
Glyma.13G119400	Late embryogenesis abundant domain-containing protein/LEA domain-containing protein	2.33 ± 0.62	0.00	1.76
Glyma.13G326400	4-hydroxy-3-methylbut-2-enyl diphosphate synthase	2.25 ± 0.16	0.00	45.50
Glyma.14G063700	HSP20-like chaperones superfamily protein	2.22 ± 0.62	0.00	0.68
Glyma.01G217700	Osmotin 34	2.21 ± 0.75	0.01	187.72
Glyma.10G064400	Embryonic cell protein 63	2.20 ± 0.33	0.00	4.44
Glyma.13G347600	Lipoxygenase 1	2.19 ± 0.98	0.03	0.14
Glyma.16G212400	Kunitz family trypsin and protease inhibitor protein	2.18 ± 0.42	0.00	946.56
Glyma.13G363300	Late embryogenesis abundant protein (LEA) family protein	2.15 ± 0.47	0.00	26.93
Glyma.03G024500	Unknown	2.13 ± 0.59	0.00	70.31
Glyma.02G234200	Peroxidase superfamily protein	2.04 ± 0.56	0.00	7.55
Glyma.04G104900	Acetyl-coa carboxylase 1	2.03 ± 0.56	0.00	13.82
Glyma.08G178100	Aldolase-type TIM barrel family protein	1.98 ± 0.44	0.00	8.82
Glyma.07G258200	NAD(P)-binding Rossmann-fold superfamily protein	1.96 ± 0.50	0.00	1.55
Glyma.16G021300	Cytochrome B5 isoform E	1.90 ± 0.42	0.00	164.67
Glyma.13G220000	Multidrug resistance-associated protein 2	1.89 ± 0.57	0.01	57.10
Glyma.05G161600	Glutathione S-transferase tau 7	1.88 ± 0.29	0.00	13.85
Glyma.08G306800	Glutathione S-transferase PHI 9	1.87 ± 0.36	0.00	68.56
Glyma.02G268000	Ethylene-forming enzyme	1.87 ± 0.58	0.02	2.02
Glyma.15G011900	Pleiotropic drug resistance 12	1.86 ± 0.34	0.00	75.60
Glyma.09G138100	AMP-dependent synthetase and ligase family protein	1.85 ± 0.14	0.00	12.93
Glyma.10G192900	Glutathione S-transferase TAU 15	1.85 ± 0.28	0.00	865.75
Glyma.13G361900	Pleiotropic drug resistance 12	1.84 ± 0.32	0.00	36.21
Glyma.13G189500	Cystatin B	1.83 ± 0.21	0.00	95.35
Glyma.09G040500	Unknown	1.80 ± 0.35	0.00	5.67
Glyma.18G164200	Uncharacterized protein family SERF	1.80 ± 0.30	0.00	577.73
Glyma.11G070600	Nmra-like negative transcriptional regulator family protein	1.80 ± 0.13	0.00	107.29
Glyma.06G310100	Alpha/beta-Hydrolases superfamily protein	1.79 ± 0.56	0.03	5.92
Glyma.11G070200	Nmra-like negative transcriptional regulator family protein	1.78 ± 0.20	0.00	748.70
Glyma.15G142900	Mitochondrial import inner membrane translocase subunit Tim17/Tim22/Tim23	1.77 ± 0.38	0.01	0.47
Glyma.09G258000	Cysteine synthase C1	1.76 ± 0.23	0.00	27.62
Glyma.03G252300	Ilityhia	1.75 ± 0.46	0.02	2.09
Glyma.15G252200	Glutathione S-transferase TAU 19	1.75 ± 0.15	0.00	12.53
Glyma.15G146600	MLP-like protein 423	1.72 ± 0.44	0.02	104.16
Glyma.13G109800	Oxophytodienoate-reductase 3	1.69 ± 0.23	0.00	9.47
Glyma.17G254200	Thioredoxin H-type 1	1.69 ± 0.34	0.01	301.45
Glyma.01G228900	Exportin 1A	1.69 ± 0.45	0.03	2.51
Glyma.15G063100	C-terminal cysteine residue is changed to a serine 1	1.68 ± 0.34	0.01	127.85
Glyma.10G277900	F1F0-atpase inhibitor protein, putative	1.68 ± 0.29	0.00	153.15
Glyma.15G115600	Auxin-responsive family protein	1.68 ± 0.35	0.01	68.67
Glyma.08G330700	Cellulose synthase like E1	1.67 ± 0.48	0.05	73.46
Glyma.18G242300	Isopentenyl diphosphate isomerase 1	1.66 ± 0.27	0.00	40.86
Glyma.08G316200	Endoribonuclease L-PSP family protein	1.65 ± 0.28	0.01	19.00
Glyma.01G106000	Glutathione S-transferase TAU 8	1.65 ± 0.26	0.00	104.18
Glyma.06G133300	Cytochrome B5 isoform E	1.64 ± 0.33	0.02	148.64
Glyma.07G117000	Atpase, F1 complex, delta/epsilon subunit	1.64 ± 0.37	0.03	87.50
Glyma.11G054500	S-adenosyl-L-methionine-dependent methyltransferases superfamily protein	1.64 ± 0.38	0.03	876.33
Glyma.04G202600	Unknown	1.63 ± 0.33	0.02	18.43
Glyma.04G021500	PAM domain (PCI/PINT associated module) protein	1.63 ± 0.36	0.03	49.87
Glyma.09G048900	Cytochrome P450, family 81, subfamily D, polypeptide 3	1.63 ± 0.33	0.02	12.47
Glyma.14G121200	Alcohol dehydrogenase 1	1.63 ± 0.32	0.01	2.04
Glyma.05G207100	Glutathione peroxidase 6	1.63 ± 0.21	0.00	284.49
Glyma.20G108500	NADH-ubiquinone oxidoreductase B18 subunit, putative	1.63 ± 0.34	0.02	179.41
Glyma.11G007600	12-oxophytodienoate reductase 2	1.62 ± 0.26	0.01	0.27
Glyma.04G221300	Manganese superoxide dismutase 1	1.62 ± 0.23	0.00	97.20
Glyma.19G214500	Unknown	1.61 ± 0.36	0.03	1.30
Glyma.05G222400	ACC oxidase 1	1.60 ± 0.34	0.03	15.93
Glyma.18G238700	Late embryogenesis abundant protein	1.60 ± 0.31	0.02	134.17
Glyma.11G099300	Ribosomal protein L12-A	1.60 ± 0.20	0.00	25.28
Glyma.19G009000	Formate dehydrogenase	1.59 ± 0.24	0.01	38.99
Glyma.17G030200	Unknown	1.59 ± 0.37	0.05	15157.50
Glyma.02G154700	Heat shock factor binding protein	1.57 ± 0.31	0.03	73.79
Glyma.02G158200	Proteinase inhibitor, propeptide	1.57 ± 0.32	0.04	129.65
Glyma.05G240100	Glutathione peroxidase 3	1.56 ± 0.20	0.00	149.18
Glyma.09G246700	Cytochrome c-2	1.56 ± 0.19	0.00	146.55
Glyma.02G104500	UDP-glycosyltransferase 73B4	1.56 ± 0.21	0.01	2.61
Glyma.09G281900	O-methyltransferase 1	1.56 ± 0.23	0.01	203.18
Glyma.04G166700	FKBP-like peptidyl-prolyl *cis*-trans isomerase family protein	1.55 ± 0.19	0.00	53.26
Glyma.03G143700	Cytochrome P450, family 93, subfamily D, polypeptide 1	1.55 ± 0.12	0.00	85.71
Glyma.07G084100	Thioredoxin-dependent peroxidase 1	1.55 ± 0.12	0.00	145.52
Glyma.02G291100	Endoribonuclease L-PSP family protein	1.55 ± 0.21	0.01	36.68
Glyma.08G179700	Peroxidase superfamily protein	1.55 ± 0.25	0.02	74.27
Glyma.11G152400	NADPH:quinone oxidoreductase	1.54 ± 0.20	0.01	129.08
Glyma.01G235600	12-oxophytodienoate reductase 2	1.53 ± 0.10	0.00	133.30
Glyma.01G126600	Disease resistance-responsive (dirigent-like protein) family protein	1.53 ± 0.16	0.00	342.60
Glyma.03G129300	S-adenosyl-L-methionine-dependent methyltransferases superfamily protein	1.52 ± 0.21	0.01	16.16
Glyma.05G109300	Little nuclei4	1.51 ± 0.16	0.00	7.88
Glyma.01G075700	Stress responsive A/B Barrel Domain	1.50 ± 0.13	0.00	0.00
**The 32 proteins with reduced accumulation in the *rn1* mutant as compared to that in the wild-type T22 line**				
Glyma.04G076300	FAD-dependent oxidoreductase family protein	0.67 ± 0.11	0.02	15.12
Glyma.20G092000	Aspartic proteinase A1	0.67 ± 0.04	0.00	107.80
Glyma.03G127200	Glycosyl hydrolases family 31 protein	0.67 ± 0.10	0.02	5.04
Glyma.05G023700	Flavin-binding monooxygenase family protein	0.66 ± 0.13	0.03	2.89
Glyma.02G070500	O-Glycosyl hydrolases family 17 protein	0.66 ± 0.09	0.01	3.06
Glyma.17G138300	Cupredoxin superfamily protein	0.66 ± 0.08	0.00	66.83
Glyma.04G092600	G-box regulating factor 6	0.65 ± 0.10	0.01	87.10
Glyma.10G245200	Ribosomal protein S3Ae	0.65 ± 0.14	0.03	187.63
Glyma.13G280200	Ribosomal protein L5 B	0.65 ± 0.15	0.04	104.07
Glyma.16G072000	2-oxoglutarate (2OG) and Fe(II)-dependent oxygenase superfamily protein	0.65 ± 0.05	0.00	105.85
Glyma.01G137700	Plant invertase/pectin methylesterase inhibitor superfamily	0.65 ± 0.12	0.02	254.65
Glyma.16G045100	Xyloglucan endotransglucosylase/hydrolase 5	0.64 ± 0.08	0.00	122.84
Glyma.03G215300	Ribosomal protein L18	0.64 ± 0.15	0.04	121.56
Glyma.07G273500	Translation elongation factor EF1B, gamma chain	0.63 ± 0.12	0.01	36.51
Glyma.08G150400	Beta glucosidase 42	0.62 ± 0.17	0.03	203.73
Glyma.09G016700	RAB gtpase homolog B1C	0.62 ± 0.14	0.02	73.60
Glyma.18G130500	Thiamin diphosphate-binding fold (THDP-binding) superfamily protein	0.60 ± 0.10	0.00	25.55
Glyma.16G043500	Apyrase 2	0.59 ± 0.09	0.00	75.40
Glyma.04G162800	Gamma subunit of Mt ATP synthase	0.59 ± 0.16	0.01	75.44
Glyma.05G149500	Amidase family protein	0.58 ± 0.13	0.00	1.09
Glyma.15G238200	Annexin 8	0.58 ± 0.12	0.00	167.60
Glyma.04G111500	Ribosomal L5P family protein	0.58 ± 0.15	0.01	150.76
Glyma.07G026400	S15/NS1, RNA-binding protein	0.55 ± 0.17	0.00	7.15
Glyma.02G048400	Flavanone 3-hydroxylase	0.55 ± 0.13	0.00	13.56
Glyma.13G199800	Annexin 8	0.53 ± 0.14	0.00	128.43
Glyma.02G224100	Histone superfamily protein	0.51 ± 0.16	0.00	69.54
Glyma.12G053800	Beta glucosidase 12	0.49 ± 0.19	0.00	144.83
Glyma.12G217300	BURP domain-containing protein	0.45 ± 0.13	0.00	2011.85
Glyma.12G217400	BURP domain-containing protein	0.44 ± 0.13	0.00	3120.32
Glyma.14G005500	Unknown	0.41 ± 0.34	0.01	26.92
Glyma.15G115000	Unknown	0.38 ± 0.20	0.00	276.49
Glyma.01G203600	Zinc-binding ribosomal protein family protein	0.33 ± 0.11	0.00	51.69

*^1^Fold Change, Mean ratio of fold changes (FC) in the protein level in rn1 mutant with that in the wild-type T322 root tissues Necrotic/Healthy root tissues) calculated from nine observations originating from three biological replications.*

*^2^Transcript Number: The Fragments Per Kilobase of transcript per Million (FPKM) mapped reads value of the genes encoded DAPs in root tissue of G. max. Williams 82 (Severin et al., 2010).*

### Functional Annotation of Differentially Accumulated Proteins

**FIGURE 2:**
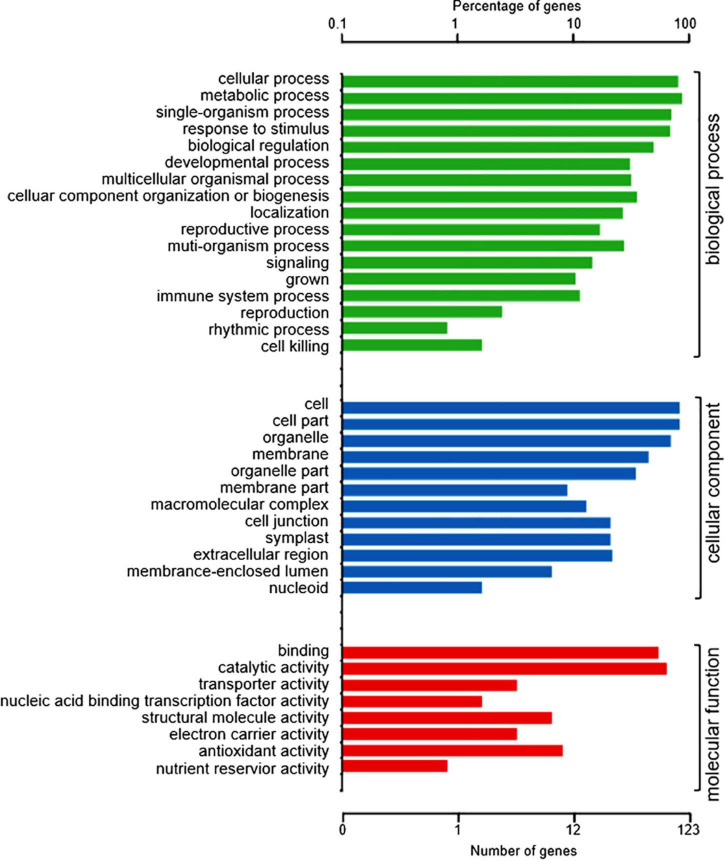
GO annotation of the 150 DAPs between necrotic (*rn1*) and healthy (T322) root tissues. The DAPs were classified into three categories: biological process, cellular component, and molecular function, based on GO terms.

To understand the role of the 150 DAPs in the spontaneous cell death process leading to root necrosis, DAPs were classified based on biological processes, cellular components and molecular functions by using the plant GO database (Harris et al., 2004). As shown in Figure 2, the DAPs were grouped into 17 basic biological processes including cellular process, metabolic process, single-organism process, response to stimulus, and biological regulation. According to cellular components, the DAPs were grouped into 12 types including cell part, cell, organelle and membrane. Based on molecular functions, the DAPs were grouped into eight classes including binding, catalytic activity, and transporter activity.

Based on the plant-specific database of Clusters of Orthologous Groups of proteins (COGs; Tatusov et al., 2003), the 150 DAPs were classified into 17 groups (Supplementary Figure 2). As expected, there were more upregulated proteins than down-regulated ones among 12 of the categories. In five categories, viz. (i) nucleotide transport and metabolism, (ii) carbohydrate transport and metabolism, (iii) intracellular trafficking, and vesicular transport, (iv) chromatin structure and dynamics, and (v) translation, ribosomal structure and biogenesis, more down-regulated proteins than upregulated proteins were represented in the *rn1* mutant compared to T322 wild-type line suggesting that transcription and translation machineries may be suppressed in the necrotic root tissues.

### Differentially Accumulated Proteins Involved in Metabolic Pathways

**FIGURE 3:**
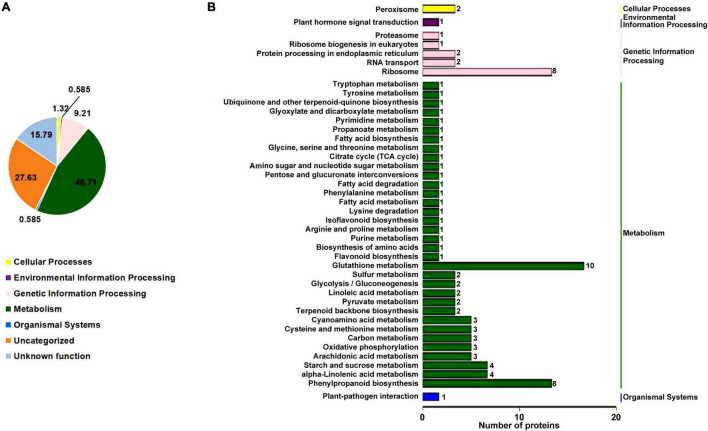
KEGG pathway enrichment analysis of the DAPs. **(A)** Based on the KEGG database pathway information, the DAPs were classified into five known categories; viz., cellular process, environmental information processing, genetic information processing, metabolism, and organismal systems. Note that 15.79% of the proteins have no known functions. **(B)** Specific pathways and number of proteins in each pathway classified under each of the five categories of the DAPs with known functions shown in **(A)** are presented. The five categories are shown on the right side of **(B)**. The number of proteins among the pathways is presented at the end of each bar.

To understand how the *rn1* mutation affects the accumulation of proteins among the known pathways, we analyzed the proteins using the Kyoto Encyclopedia of Genes and Genomes (KEGG) pathway database. This database is an ideal tool to predict the gene function by linking genomic information with higher order functional information of cellular processes including metabolism, membrane transport, signal transduction and cell cycle (Kanehisa et al., 2017). The overall enrichment of the 150 DAPs is shown in Figure 3A. The majority of the DAPs are involved in metabolism (46.71%). Accumulation patterns of a large number of uncharacterized soybean proteins (27.63%) and with unknown function (15.79%) are also affected by the *rn1* mutation. DAPs were classified to 42 pathways under five categories with known functions (Figure 3B). Several of the proteins are involved in primary and secondary metabolism. Pathways enriched among the DAPs include glyoxylate and dicarboxylate metabolism, propanoate metabolism, cyanoamino acid metabolism, sulfur metabolism, arachidonic acid metabolism, alpha-linolenic acid metabolism, linolenic acid metabolism, carbon fixation in photosynthetic organ, terpenoid backbone biosynthesis, isoflavonoid biosynthesis, and phenylpropanoid biosynthesis. DAPs were mapped to pathways using MapMan bin codes (Supplementary Figure 3). The 21 DAPs were found to be associated with RNA-protein syntheses pathways (Supplementary Figure 4).

### Differentially Accumulated Proteins Involved in Biotic and Abiotic Stress Pathways

**FIGURE 4:**
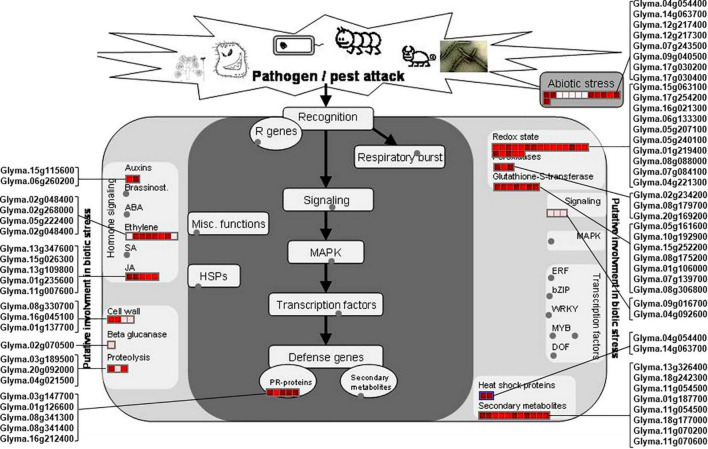
DAPs involved in pathways induced by biotic and abiotic stresses. The DAPs involved in plant biotic and abiotic stresses were mapped using MapMan software. Proteins involved into the biotic and abiotic stresses are presented in red font. The scale represented the fold changes of DAPs, which is presented in the upper left corner of the figure.

The *rn1* mutation enhances tolerance to the oomycete pathogen *P. sojae* in soybean (Kosslak et al., 1997). Plants respond to pathogen attacks by a rapid change in gene expression levels leading to accumulation of biotic stress-related defense proteins and metabolites or compounds. MapMan was used to analyze the 150 DAPs to determine which DAPs are involved in pathways that are activated by biotic and abiotic stresses. Among the 150 DAPs, 53 and eight DAPs were found to be involved in biotic and abiotic stresses, respectively, (Figure 4).

Of the identified biotic stress-related proteins, five DAPs (Glyma.01g126600, Glyma.03g147700, Glyma.08g341300, Glyma.08g341400, and Glyma.16g212400) are pathogenesis-related (PR) proteins are highly induced in the *rn1* mutant as compared to that in T322. They are also expressed in roots and could be involved in enhancing tolerance to *P. sojae* (Figure 4).

Several DAPs responsive to biotic stress are involved in hormone signaling, redox reaction, signal transmission, cell wall modification, and synthesis of secondary metabolites observed in plant–pathogen interaction process. For example, 11 DAPs, auxins-associated proteins (Glyma.15g115600 and Glyma. 06g260200), ethylene-associated proteins (Glyma.02g048400, Glyma.02g268000, Glyma.05g222400, and Glyma.02g048400), and JA-associated proteins (Glyma.13g347600, Glyma. 15g026300, Glyma.13g109800, Glyma.01g235600, and Glyma.11g007600), participate in hormone-regulated defense signaling pathways and mediate the biosynthesis of plant defense compounds (Figure 4).

### Identification of Phosphorylated Proteins

Protein phosphorylation is a key mechanism involved in regulating protein functions. In this study, we identified a total of 573 phosphopeptides of 146 proteins through LC-MS/MS analysis (Supplementary Table 1). Of the 573 phosphopeptides, only 234 were unique phosphopeptides. Of the 234 peptides, only 165 peptides were identified. GO analysis of the phosphorylated 146 proteins assigned the phosphoproteins (PhosPs) to 19 classes based on biological processes, 11 based on cellular components, and 10 based on molecular functions (Supplementary Figure 5). Based on molecular functions, PhosPs were grouped into many classes of proteins with binding and catalytic activity, transporter activity, nucleic acid binding, signal transducer activity, electron carrier activity, molecular transducer activity, molecular function regulator and antioxidant activity. PhosPs with antioxidant activity could be involved in conferring disease resistance.

Among the 146 PhosPs, 143 proteins displayed detectible expression levels in root (Supplementary Table 1). Only two of 143 proteins were found to be significantly changed in their steady state levels (≤0.67-FC, *p*-value ≤ 0.05) due to the *rn1* mutation. One is Phosphoglucomutase/phosphomannomutase family protein (Glyma.05G237000.1), and the other one is an unknown protein (Glyma.16G037900.1). The levels of phosphorylation for these two differentially accumulated phosphoproteins are significantly reduced in the *rn1* mutant root tissues as compared to that in the healthy root tissues of T322.

### Phosphorylation Levels of Phosphoproteins Affected by the *rn1* Mutation

We analyzed the proteomic and phosphoproteomic data to investigate how the *rn1* mutation contributes to the phosphorylation statuses of the PhosPs. For this study, we selected 88 PhosPs that contain phosphopeptides with significantly different levels of phosphorylation between the root tissues of *rn1* and T322. For each PhosP, nine comparisons were made for “levels of phosphorylation” (A) and “levels of PhosP” (B); i.e., each of the three replications of the *rn1* root sample was compared to each of the three root samples of the T322 wild-type plants for phosphorylation levels (A = *rn1*/T322) or steady state protein levels of PhosP (B = *rn1*/T322). The “A” values from nine comparisons were then compared to respective nine “B” values from nine comparisons of PhosP levels to obtain nine A/B ratios for each of the 88 PhosPs.

**FIGURE 5:**
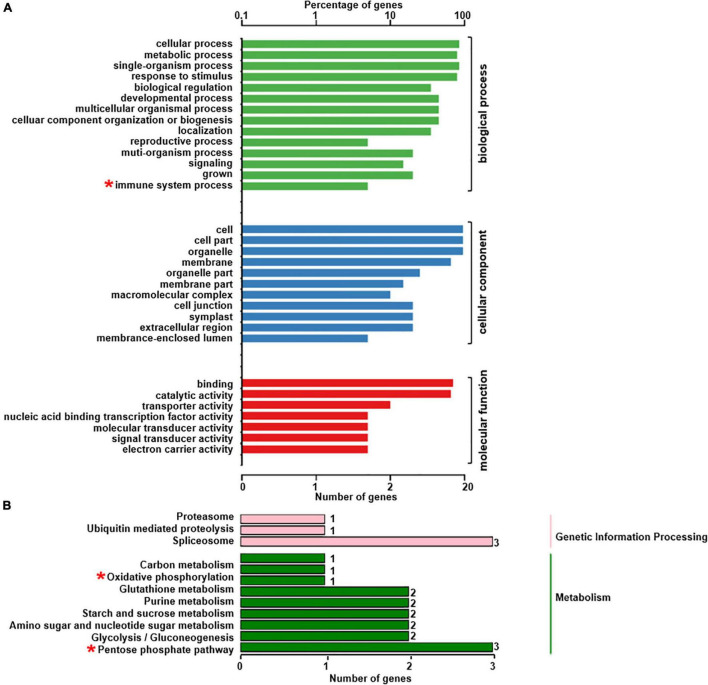
GO and KEGG analysis for 22 phosphoproteins that were detectible (Table 2). **(A)** Based on GO terms, the phosphoproteins were classified into three categories: biological process, cellular component, and molecular function. **(B)** Specific pathways and number of phosphoproteins in each pathway classified under each of the two categories. Some phosphoproteins are significantly involved in immune system process, oxidative phosphorylation and pentose phosphate pathway, as indicated by “*”.

The phosphoproteomics analysis identified 22 phosphopeptides, the levels of phosphorylation of which were significantly different between *rn1* and T322 lines (Table 2). Among the 22 phosphopeptides, two showed enhanced phosphorylation levels, and 20 showed reduced phosphorylation levels in the *rn1* compared to the wild-type 322. The PhosPs carrying these 22 phosphopeptides were grouped into 14 classes based on basic biological processes, 11 based on cellular components and seven classes based on molecular functions (Figure 5A). In particular, some of these PhosPs are involved in the immune response process.

**TABLE 2:** Differential phosphorylation levels of 22 phosphopeptides that are detectible.

Phosphoprotein	Annotation	Phosphopeptide	*n*	Mean (A/B)	Mean (1-A/B)	SE	*p-v*alue

The two phosphopeptides that showed enhaned phosphorylation levels in the *rn1* mutant as compaed to the wild-type-T322							
Glyma.15G227300	Nuclear factor Y, subunit C11	AIGDDGND 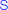 DEEAKR	1	1.56	−0.56	0.15	0.00
Glyma.07G159600	Unknown	GEE 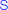 DSGNDHGSVK	1	1.23	−0.23	0.07	0.01
**The 20 phosphopeptides that showed reduced phosphorylation levels in the *rn1* mutant as compaed to the wild-type-T322**							
Glyma.01G175100	U1 small nuclear ribonucleoprotein-70K	EQQQ 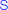 RSEEPR	2	0.88	0.12	0.04	0.01
Glyma.02G216000	Ubiquitin-protein ligase 1	SLDVEIG 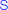 ADGHDDGGER	1	0.73	0.27	0.09	0.02
Glyma.03G167100	Nuclear transport factor 2 (NTF2) family protein with RNA binding (RRM-RBD-RNP motifs) domain	GQPVLSAAPQYAPQH 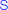 FK	5	0.74	0.26	0.11	0.05
Glyma.03G252300	ILITYHIA	ALLEGG 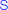 DDEGSSTEAHGR	10	0.65	0.35	0.07	0.00
Glyma.04G200100	Unknown	QW 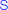 GGSSSTGSSSPAMSPAHPQSR	1	0.77	0.23	0.10	0.04
Glyma.05G005500	DEAD box RNA helicase family protein	GHGASDAGAGL 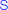 PESYR	2	0.74	0.26	0.07	0.01
Glyma.05G237000	Phosphoglucomutase/phosphomannomutase family protein	ATGAFILTA 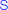 HNPGGPHEDFGIK	17	0.80	0.20	0.06	0.01
Glyma.06G255200	Adenine nucleotide alpha hydrolases-like superfamily protein	IHHPA 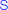 PR	6	0.66	0.34	0.07	0.00
Glyma.08G044100	Phosphoglucomutase/phosphomannomutase family protein	ATGAFILTA 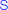 HNPGGPNEDFGIK	15	0.70	0.30	0.06	0.00
Glyma.08G233300	Metacaspase 5	GEGQQHSGSGSGFGLS 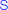 FLR	3	0.73	0.24	0.07	0.01
Glyma.08G233500	Metacaspase 4	GEEEQSGSGFGFS 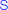 FLHR	8	0.76	0.23	0.06	0.00
Glyma.09G056300	H(+)-ATPase 2	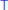 LHGLQPPETSNIFNEK	7	0.76	0.24	0.06	0.00
Glyma.10G239100	Arginine/serine-rich splicing factor 35	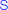 PQNGHGSSR	4	0.76	0.24	0.06	0.00
Glyma.11G078100	Regulatory particle non-ATPase 10	GDEQQA 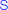 SQHATMTER	2	0.73	0.27	0.07	0.00
Glyma.13G361900	Pleiotropic drug resistance 12	GLLTA 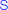 HGVANEIDVSDLGTQER	2	0.64	0.36	0.05	0.00
Glyma.15G011900	Pleiotropic drug resistance 12	GLLTA 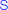 HGVANEIDVSDLGIQER	8	0.68	0.32	0.03	0.00
Glyma.16G037900	Unknown	HEHGHDS 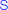 SSSDSD	1	0.64	0.36	0.06	0.00
Glyma.16G063200	Glucose-6-phosphate dehydrogenase 6	RS 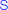 FGSESPLAR	6	0.79	0.21	0.08	0.03
Glyma.17G254200	Thioredoxin H-type 1	HASAVAAASS 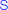	3	0.66	0.34	0.06	0.00
Glyma.18G225900	S-adenosyl-L-methionine-dependent methyltransferases superfamily protein	GHSNSSSSS 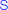 PSK	3	0.83	0.17	0.07	0.04

*Phosphopeptide, the predicted phosphorylation sites are shown in bold blue font. n, Number of peptide-spectrum match for each peptide. A/B, Mean ratio of the phosphorylation level of a peptide in rn1 mutant with that in the wild-type T322 root tissues calculated from nine observations originating from three biological replications in each group. The phosphorylation level of a peptide was standardized against its accumulated level among the nine determinations from three biological replications. 1-A/B, Mean of the deviations of the A/B ratios from 1, with no change in phosphorylation level of a phosphopeptide in rn1 mutant with that in the wild-type T322 root tissues, calculated from nine observations originating from three biological replications.*

**FIGURE 6:**
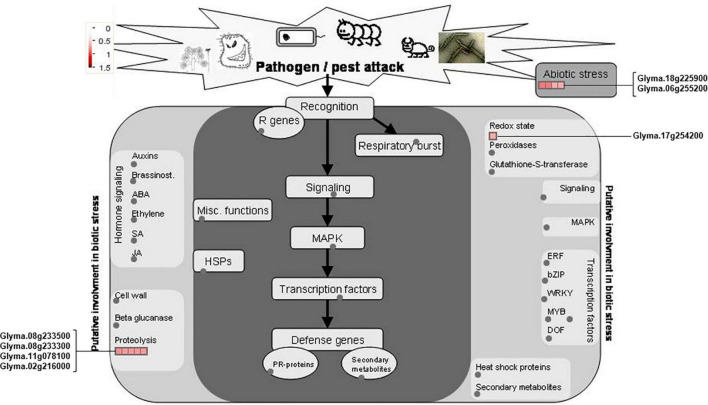
MapMan analysis of 22 PhosPs listed in Table 2. Seven phosphoproteins were induced by biotic and abiotic stresses are shown on both sides of the map.

**FIGURE 7:**
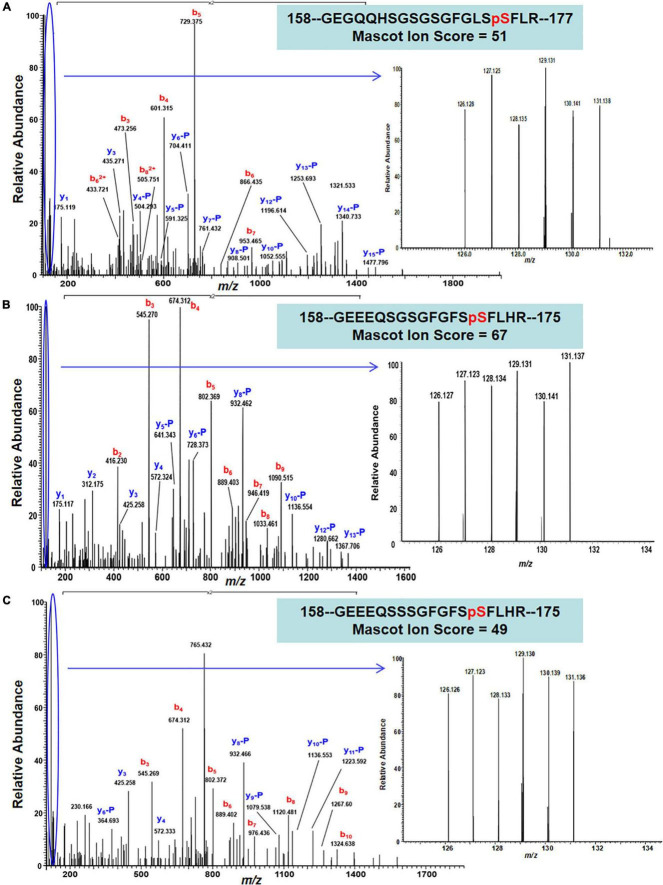
MS/MS spectra show confident identifications of three representative phosphopeptides/sites that were labeled by TMT6-plex from three Metacaspase proteins. **(A)** An MS/MS spectrum of a triply charged ion at m/z 768.689^3+^ confidently identifying a TMT-labeled tryptic peptide with S174 phosphorylation site from Metacaspase 5 protein (Glyma.08G233300) between necrotic and healthy roots; **(B)** an MS/MS spectrum of a triply charged ion at m/z 756.327^3+^ confidently identifying a TMT-labeled tryptic peptide with S171 phosphorylation site from Metacaspase 4 protein (Glyma.08G233500), and **(C)** an MS/MS spectrum of a triply charged ion at m/z 766.330^3+^ confidently identifying a TMT-labeled tryptic peptide with S171 phosphorylation from Metacaspase 4 protein (Glyma.15G219100) between necrotic and healthy roots. The inset of each panel shows the expanded view of relative intensity of 6 TMT reporter ions for determining the abundance changes of the S174, S171, and S171 phosphopeptides in necrotic root triplicate (126, 127, and 128) versus 3 healthy roots (129,130, and 131).

The 22 PhosPs were enriched into 12 pathways (Figure 5B). It is clear that some proteins are related to the oxidative phosphorylation and pentose phosphate pathway, viz. (i) H^+^ ATPase (Glyma.09G056300) involved in oxidative phosphorylation (Supplementary Figure 6); (ii) phosphoproteins related to the pentose phosphate pathway enzymes are glucose-6-phosphate dehydrogenase 6 (Glyma.16g063200 and Glyma.19g082300), phosphoglucomutase/phosphomannomutase (Glyma.08g044100 and Glyma.05g237000) family protein, (Supplementary Figure 7). MapMan analysis revealed that seven of the 22 PhosPs are induced by biotic and abiotic stresses (Figure 6). Especially, phosphorylation levels of two highly similar type II metacaspases (Glyma.08G233300 and Glyma.08G233500) were significantly reduced in the *rn1* mutant root tissues as compared to that in the healthy root tissues of T322, suggesting that the dephosphorylation or reduced phosphorylation of type II metacaspases may contribute toward initiating spontaneous cell death observed in roots of the lesion mimic *rn1* mutant (Supplementary Figure 8). The phosphopeptides of the Glyma.08G233300 and Glyma.08G233500 with significant differences in phosphorylation levels (Table 2 and Supplementary Table 2), and Glyma.15G219100.1 (Table 3 and Supplementary Table 2) with statistically non-significant reduced phosphorylation level were localized to the C-terminal region of the P20 caspase-like domain (Supplementary Figure 9). The MS/MS spectra of the identified phosphopeptides from all three type II metacaspases (Glyma.08G233300, Glyma.08G233500, and Glyma.15G219100) along with their quantitative changes between the *rn1* mutant and wild-type 322 lines are shown in Figure 7.

**TABLE 3:** Differential phosphorylation of 24 phosphopeptides that are less abundant.

Protein accession	Annotation	Phosphopeptide	*n*	FC	1-FC	SE	*p-*value
**The three phosphopeptides showing enhanced phosphorylation in the *rn1* mutant as compared to T322**							
Glyma.10G257200	Unknown	SY**S**HSESSNR	2	1.48	-0.48	0.18	0.03
Glyma.16G212900	Yellow stripe-like 7	ENVLPVADQD**S**PSNSHLSYDDQR	1	1.28	-0.28	0.10	0.02
Glyma.07G237900	RNA binding;abscisic acid binding	GGG**S**PDHLDGGNFAK	1	1.28	-0.28	0.12	0.05

**The 21 phosphopeptides showing reduced phosphorylation in the *rn1* mutant as compared to T322**							
Glyma.10G210700	Phosphoglucosamine mutase family protein	NEAFLCPADGSIMITA**S**HLPFNR	1	0.89	0.11	0.04	0.02
Glyma.12G226300	Protein kinase superfamily protein	GQHDESDSPQ**T**PR	5	0.87	0.13	0.05	0.04
Glyma.18G195500	RING/U-box superfamily protein	GPQRPVF**S**PGSNSQQHDLEDK	1	0.86	0.14	0.06	0.04
Glyma.10G247200	Protein of unknown function (DUF1677)	SDL**S**SSSSSSSPSSSK	1	0.86	0.14	0.06	0.03
Glyma.09G204100	Polyketide cyclase/dehydrase and lipid transport superfamily protein	AESSAS**T**SEPDSDDNHHR	1	0.85	0.15	0.04	0.00
Glyma.01G036700	Sterile alpha motif (SAM) domain-containing protein	GI**S**PQR	1	0.84	0.16	0.07	0.04
Glyma.08G243000	UDP-glucose 6-dehydrogenase family protein	FDWDHPIHLQPT**S**PTTVK	9	0.83	0.17	0.07	0.04
Glyma.08G243000	UDP-glucose 6-dehydrogenase family protein	FDWDHPIHLQPTSP**T**TVK	12	0.81	0.19	0.06	0.02
Glyma.18G242700	Protein phosphatase 2C family protein	SPHPN**S**PNSSSFR	1	0.79	0.21	0.08	0.03
Glyma.04G052900	Eukaryotic translation initiation factor 4A1	VHACVGG**T**SVR	4	0.76	0.24	0.06	0.00
Glyma.15G261200	Protein kinase superfamily protein with octicosapeptide/Phox/Bem1p domain	VP**S**VEHNQNLTSK	2	0.75	0.25	0.09	0.02
Glyma.10G034800	BET1P/SFT1P-like protein 14A	ASSLYSSS**S**SHEIDEHDNEQALDGLQDR	1	0.75	0.25	0.10	0.03
Glyma.05G011600	Leucine-rich repeat protein kinase family protein	GSEGH**T**PPPESR	1	0.75	0.25	0.09	0.02
Glyma.10G034800	BET1P/SFT1P-like protein 14A	RL**S**GDINEEVDSHNR	1	0.73	0.27	0.07	0.00
Glyma.07G070900	Unknown	HD**S**PSEDVSHR	2	0.71	0.33	0.07	0.00
Glyma.15G056100	Homologue of NAP57	HESTD**S**PVAVPAK	1	0.70	0.30	0.07	0.00
Glyma.08G297500	IQ-domain 28	GHGQG**S**PR	1	0.70	0.30	0.06	0.00
Glyma.09G204100	Polyketide cyclase/dehydrase and lipid transport superfamily protein	AESSAST**S**EPDSDDNHHR	1	0.67	0.34	0.06	0.00
Glyma.03G163600	ENTH/VHS/GAT family protein	GRDEPVDMAGGN**S**PHVPYASESYVDAPER	1	0.66	0.34	0.07	0.00
Glyma.10G255700	Pre-mRNA-processing protein 40B	HSSGHE**S**DEGR	1	0.62	0.38	0.08	0.00
Glyma.10G201500	Unknown	GEGRH**S**DDGNQ	1	0.55	0.45	0.10	0.00

*Phosphopeptide, the predicted phosphorylation sites are shown in bold black font. n, Number of peptide-spectrum match for a phosphopeptide. FC, Mean ratio of fold changes (FC) in the phosphorylation level of a peptide in rn1 mutant with that in the wild-type T322 root tissues calculated from nine observations originating from three biological replications. 1-FC, Mean of the deviations of the FC from 1, with no change in phosphorylation level of a phosphopeptide in rn1 mutant with that in the wild-type T322 root tissues, calculated from nine observations originating from three biological replications.*

We have identified 24 less abundant PhosPs containing phosphopeptides that exhibited significantly different phosphorylation levels between the *rn1* mutant and T322 wild-type root tissues (Table 3 and Supplementary Table 2). The steady state levels of these peptides could not be detected because of their low abundance. Therefore, phosphorylation levels of the 24 phosphopeptides between *rn1* mutant and T322 lines could not be compared as in for 22 PhosPs (Table 2), and are considered as candidates for future studies. These phosphopeptides were classified into 15 biological processes, 11 cellular components, and three molecular functions based on GO terms (Supplementary Figure 10A). The 24 phosphopeptides can be classified into nine pathways (Supplementary Figure 10B). MapMan analysis of the 24 phosphopeptides revealed that four proteins (Glyma.08G243000, Glyma.18G195500, Glyma.05G011600, and Glyma.08G297500) involved in biotic and abiotic stresses pathways (Supplementary Figure 11).

## Discussion

Lesion mimic mutants have been isolated from multiple plant species (Lorrain et al., 2003) and are ideal for studying the role of cell death in defense responses and molecular mechanisms regulating the PCD process and defense responses in plants (Jones and Dangl, 1996; Mittler and Lam, 1996; Bruggeman et al., 2015). Barley Mlo protein is a negative regulator of immunity against the powdery mildew pathogen *Erysiphe graminis* f. sp. *hordei*. Loss of function *mlo* mutant exhibits broad spectrum resistance against all isolates of *E. graminis* f. sp. *hordei* in barley (Jorgensen and Mortensen, 1977). Powdery mildew disease appears in many crop species and *Mlo* is conserved across plant species. *Mlo* orthologues have been mutated in several plant species including apple, grapevine, cucumber, pepper, tomato, melon, rose, wheat, *Arabidopsis*, and tobacco to enhance resistance against the powdery mildew pathogens (Kusch and Panstruga, 2017).

Thirty recessive *rn* mutant alleles were identified and mapped to the same locus on soybean chromosome 18 (Palmer et al., 2008). The *rn1* mutant shows progressive browning of the root system over time with visible necrotic phenotype appearing between 3 and 5 days after germination (Kosslak et al., 1996, 1997). The *rn1* mutant showed enhanced accumulation of phytoalexins glyceollin and pathogenesis-related proteins; and as a result, enhanced tolerance of the mutant to *P. sojae* is observed (Graham and Graham, 1991; Kosslak et al., 1997). Here we have conducted quantitative proteomics and phosphoproteomics studies of the roots of near-isogenic *rn1* and wild-type 322 plants to investigate the genetic mechanisms regulated by the *Rn1* gene. We detected 150 DAPs, including 118 significantly up-regulated and 32 downregulated proteins in the necrotic root tissues as compared to that in the tissues of healthy roots of the progenitor T322 line.

Analyses of 150 DAPs by applying several bioinformatics tools such as GO annotation, COG, KEGG pathway, MapMan provided a better understanding of the biological or genetic pathways that are affected by the *rn1* mutation. As expected, accumulation of many biotic stress-responsive proteins including PR proteins observed in earlier studies was increased in the *rn1* mutant (Kosslak et al., 1996, 1997). A majority of the proteins/enzymes are involved in metabolic pathways (46.71%) and greatly affected by the *rn1* mutation (Figure 3). In addition, levels of enzymes involved in the secondary metabolic pathways for synthesis of defense compounds are elevated in the *rn1* mutant. Analysis based on COGs revealed that although the secondary metabolic pathways for synthesis of defense compounds are elevated, and the accumulation of proteins or enzymes involved in translation, ribosomal structure and biogenesis is reduced in the *rn1* mutant indicating that the translation machinery may be compromised in the necrotic tissues due to onset spontaneous cell death. It was observed that 27.63% of the DAPs affected by the *rn1* mutation are currently uncharacterized (Figure 3).

This study was undertaken to reveal the molecular basis of cell death pathway mediated by Rn1. Caspase-like activities have been observed in plants during PCD consistently since the first report of the enzyme in 1998 (del Pozo and Lam, 1998). Caspases, cysteine-dependent aspartate specific proteases, are central components that mediate apoptosis, an equivalent process of plant PCD in animals, in response to various stimuli (Parrish et al., 2013). Soon after caspases were identified as major regulators of cell death in mammals, a hunt for homologous peptidases in other kingdoms led to identification of caspase-like proteins, metacaspases, from plants, fungi, and protozoa (Uren et al., 2000).

In certain plant-pathogen interactions, the PCD seems to be mediated by metacaspases (Fagundes et al., 2015). Metacaspases are divided into two types, type I and type II, according to the structural feature of the linker between the P20-like and P10-like domains (Supplementary Figure 9). The type II metacaspases have a much longer linker between the two subunits (Carmona-Gutierrez et al., 2010). Many studies have shown that type II metacaspases play an important role in regulating PCD during biotic and abiotic stresses. In *Arabidopsis*, *AtMC8* was identified as a positive mediator of cell death induced by ultraviolet C radiation and oxidative stress (He et al., 2008). Two *AtMCP2d* mutants (*mcp2d-1* and *mcp2d-3*) exhibited reduced sensitivity to PCD-inducing mycotoxin fumonisin B1 as well as oxidative stress inducers, whereas *AtMCP2d* over-expressors were more sensitive to these agents, and exhibited accelerated cell-death progression (Watanabe and Lam, 2011). Knocking down the expression of the type II *Triticum aestivum* metacaspase gene *TaMCA4* through virus-induced gene silencing compromised the immunity of the “Suwon11” wheat line against the avirulent race of *Puccinia striiformis* f. sp. *tritici* with reduced necrotic symptoms at the infection sites (Wang et al., 2012). A tomato metacaspase gene is upregulated during PCD induced in *Botrytis cinerea*-infected leaves (Hoeberichts et al., 2003). A large number of studies suggested that plant type II metacaspases facilitate the development of the morphological features that are characteristics of various classes of the PCD (Ahmad et al., 2012).

In this study, we were able to identify 146 PhosPs that carry phosphopeptides. Of the 146 PhosPs, the phosphorylation levels were significantly changed between *rn1* and T322 lines for only 22 PhosPs (Table 2). Among the 22 PhosPs, some are involved in the oxidative phosphorylation, pentose phosphate and plant hormone signaling pathways based on KEGG classification. MapMan analysis suggested that some of the proteins are biotic and abiotic stress-related. A family of two highly similar type II metacaspases was detected (Supplementary Figures 8, 9). The phosphorylation levels of these II metacaspases were significantly reduced in the *rn1* mutant root tissues as compared to that in the healthy root tissues of T322, suggesting that the dephosphorylation or reduced phosphorylation of type II metacaspases may contribute toward initiating spontaneous cell death observed in the lesion mimic *rn1* mutant. The three phosphopeptides identified from three Type II metacaspases, respectively, are highly similar and localized to the same C-terminal region of the P20 caspase-like domain (Supplementary Figure 9). Earlier, we identified a type II metacaspase (metacaspase 5, Glyma.08G233300; Table 2) as an interactor of the coiled-coil nucleotide-binding site leucine-rich repeat region (NB-LRR) *Phytophthora* resistance Rps1-k-2 protein (Gao et al., 2005; Gao, 2006; Gao and Bhattacharyya, 2008; Baskett, 2012). The same protein has been identified in this study as PhosPs, phosphorylation level of which is significantly reduced in the *rn1* mutant as compared to the wild-type T322 line.

In plants under stress conditions, metacaspase type I positively regulates HR cell death induced by activated NB-LRR proteins following recognition of pathogen effectors. LSD1 negatively regulates the spread of cell death presumably by binding to type I metacaspase. The type II metacaspases with reduced phosphorylation levels may represent an inactive form with no or highly reduced negative regulatory role on metacaspase type I in the *rn1* mutant roots resulting in spontaneous root necrosis (Fagundes et al., 2015). This study showed that phosphorylation of at least two type II metacaspases at least partly by Rn1 most likely suppresses the cell death process, which becomes spontaneously active in the *rn1* mutant in absence of the functional Rn1 protein. The third type metacaspase identified in this study also showed a trend of reduced phosphorylation level in absence of Rn1 protein. In all three metacaspases identified in this study showed a serine residue for phosphorylation located in the P20-like domain (Figure 7; Supplementary Figure 9; and Table 2).

The phosphorylation levels of these II metacaspases were significantly reduced in the *rn1* mutant root tissues as compared to that in the healthy root tissues of T322, suggesting that the dephosphorylation or reduced phosphorylation of type II metacaspases could contribute toward initiating spontaneous cell death observed in the root lesion mimic *rn1* mutant.

## Conclusion

In this study, we conducted proteomics and phosphoproteomics of the necrotic root tissues of the *rn1* mutant and healthy root tissues of the progenitor T322 line, and detected 150 DAPs and 22 potential phosphoproteins involved in manifestation of the root necrotic phenotype. These candidate proteins will facilitate discovering the molecular mechanisms involved in the cell death process in soybean roots. We have identified two type II metacaspases (Glyma.08G233300 and Glyma.08G233500), the phosphorylation levels of which were reduced in *rn1* as compared to T322, implying that Rn1 either directly or indirectly phosphorylates type II metacaspases to negatively regulate the cell death process in soybean roots.

## Data Availability Statement

The datasets presented in this study can be found in online repositories. The names of the repository/repositories and accession number(s) can be found below: The mass spectrometry proteomics data have been deposited to the ProteomeXChange consortium *via* the PRIDE partner repository with the dataset identifier PXD032226.

## Author Contributions

FW analyzed the data, wrote the manuscript, and prepared the figures. PD and NP performed the experiments. SZ contributed to proteomic and phosphoproteomics data acquisition and analysis, manuscript editing. RB prepared the mass spectrometry figures. MB designed the experiments and edited and finalized the manuscript. All authors contributed to the article and approved the submitted version.

## Conflict of Interest

The authors declare that the research was conducted in the absence of any commercial or financial relationships that could be construed as a potential conflict of interest.

## Publisher’s Note

All claims expressed in this article are solely those of the authors and do not necessarily represent those of their affiliated organizations, or those of the publisher, the editors and the reviewers. Any product that may be evaluated in this article, or claim that may be made by its manufacturer, is not guaranteed or endorsed by the publisher.

## Footnotes

1
https://phytozome.jgi.doe.gov/pz/portal.html

2
https://github.com/tanghaibao/GOatools

3 https://www.plabipd.de/portal/mercator-sequence-annotation; http://www.kegg.jp/; or http://www.genome.jp/kegg/

4
https://mapman.gabipd.org/
