# Supplementary material for: A Phosphoproteomics Study of the Soybean root necrosis 1 Mutant Revealed Type II Metacaspases Involved in Cell Death Pathway
Source: Front Plant Sci. 2022 Jul 19;13:882561. doi: 10.3389/fpls.2022.882561 (PMC9344878; doi:10.3389/fpls.2022.882561)
Supplement: Supplementary file 1 [file Data_Sheet_1.docx]

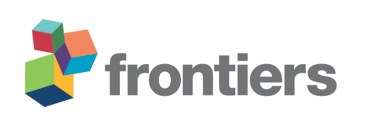


Supplementary Material

## Supplementary Figures


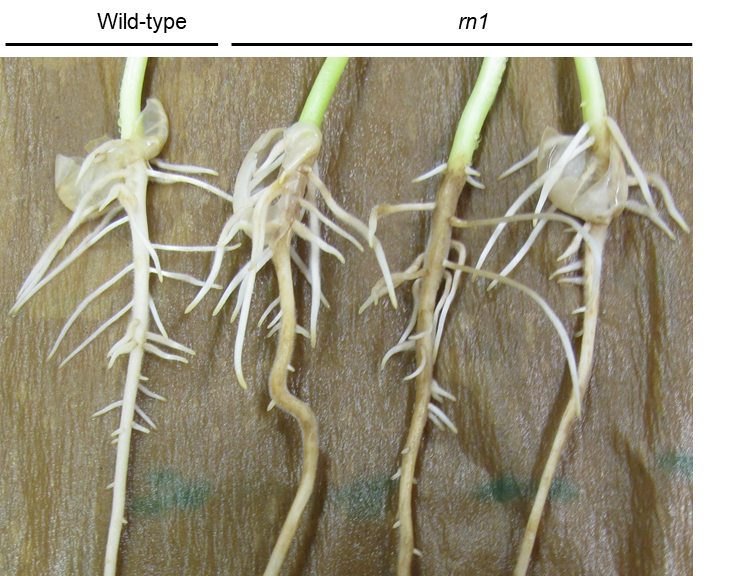


**Supplementary Figure 1** The necrotic root phenotype of the *rn1* (T328H) mutant.


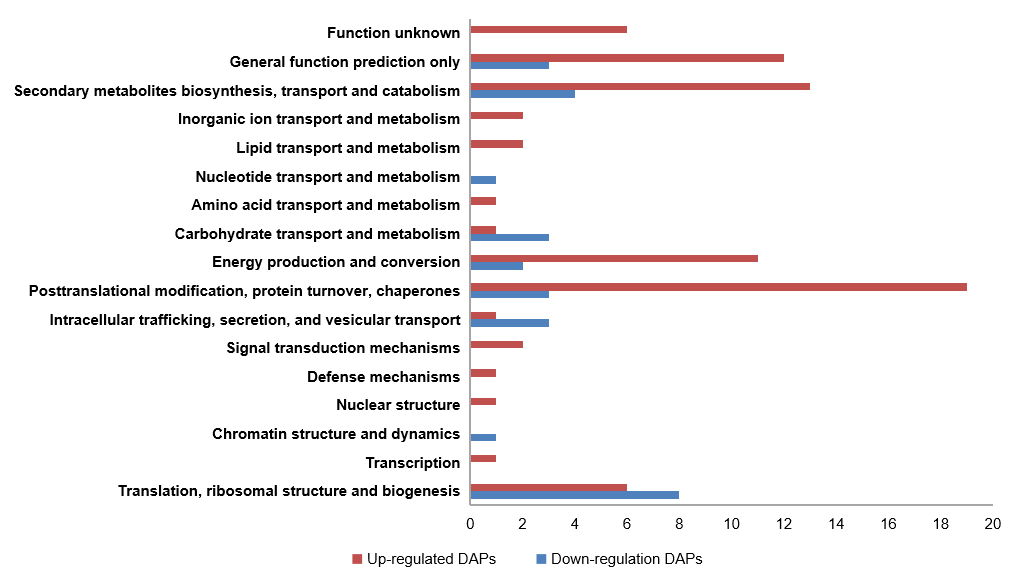


**Supplementary Figure 2** Categories of up- and down-regulated DAPs based on plant COGs database.

**Supplementary Figure 3** The DAPs that were mapped using MapMan bin codes to metabolic pathways affected by the *rn1* mutation. (A) The squares in the functional category indicate the levels of up- or down-regulation of the DAPs in the *rn1* mutant as compared to that in T322 wild-type control, while circles represent those proteins, the accumulation levels of which are not affected by the *rn1* mutation. The ID of DAPs involved in metabolic pathways are presented in the Supplement Table S2. (B) Ten DAPs are involved in secondary metabolism pathway.


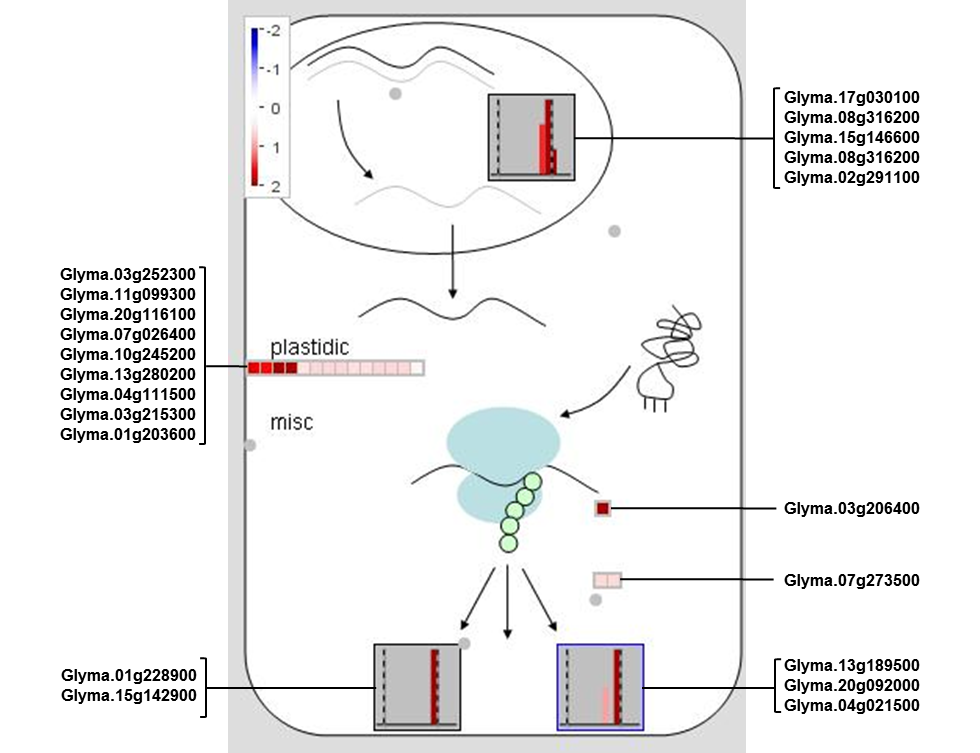


**Supplementary Figure 4** MapMan analysis of the DAPs revealed the proteins/enzymes that are involved in RNA-protein metabolism. The 21 DAPs (in black font) related to RNA-protein synthesis pathways are shown.


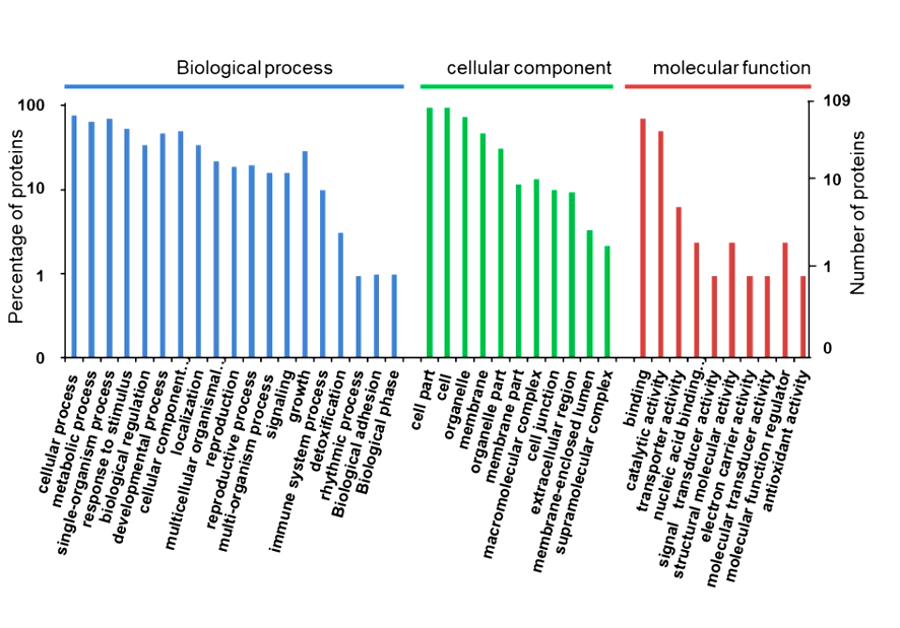


**Supplementary Figure 5** Classification of the phosphorylated proteins based on GO terms. The 146 identified phosphorylated proteins were classified based on the biological processes, cellular components and molecular functions


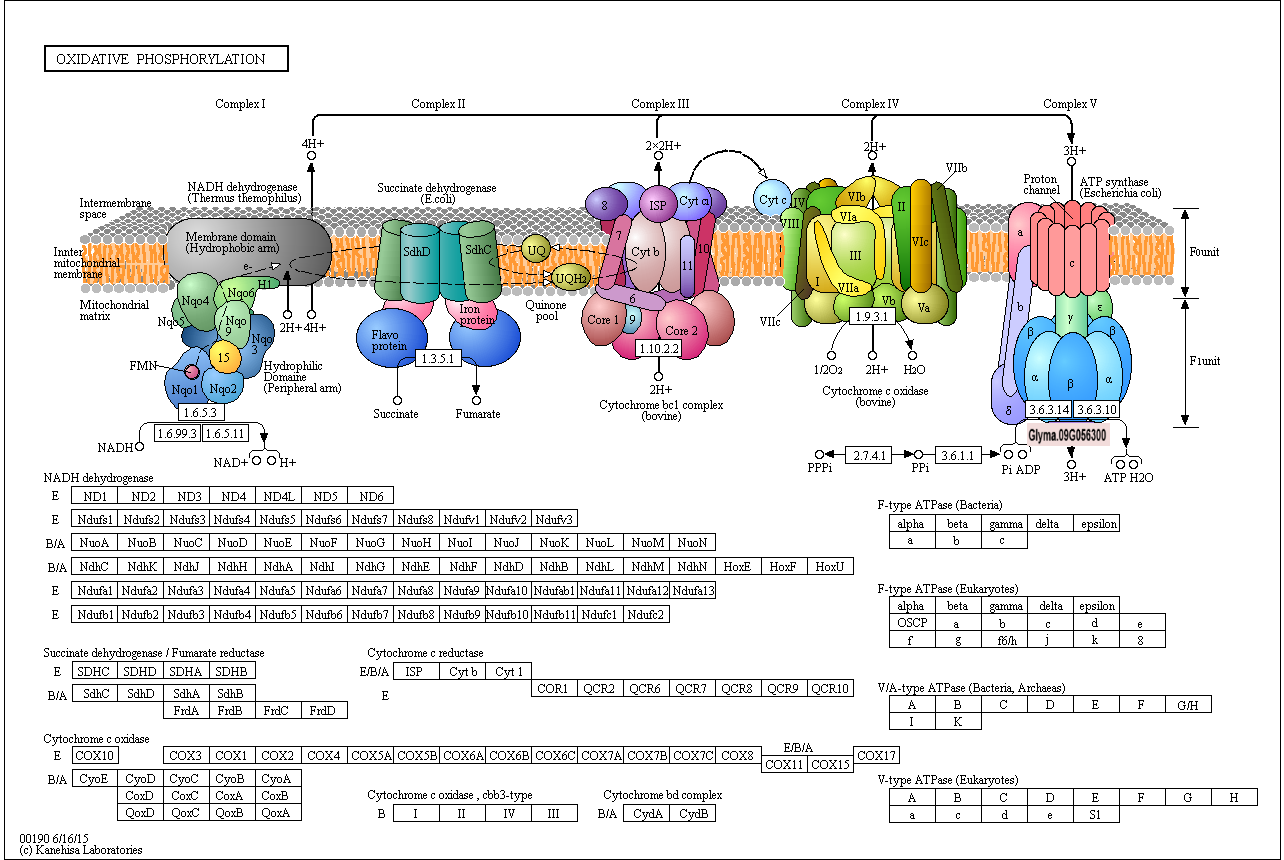


**Supplementary Figure 6** Glyma.09G056300 encoding H+-ATPase was involved into oxidative phosphorylation.


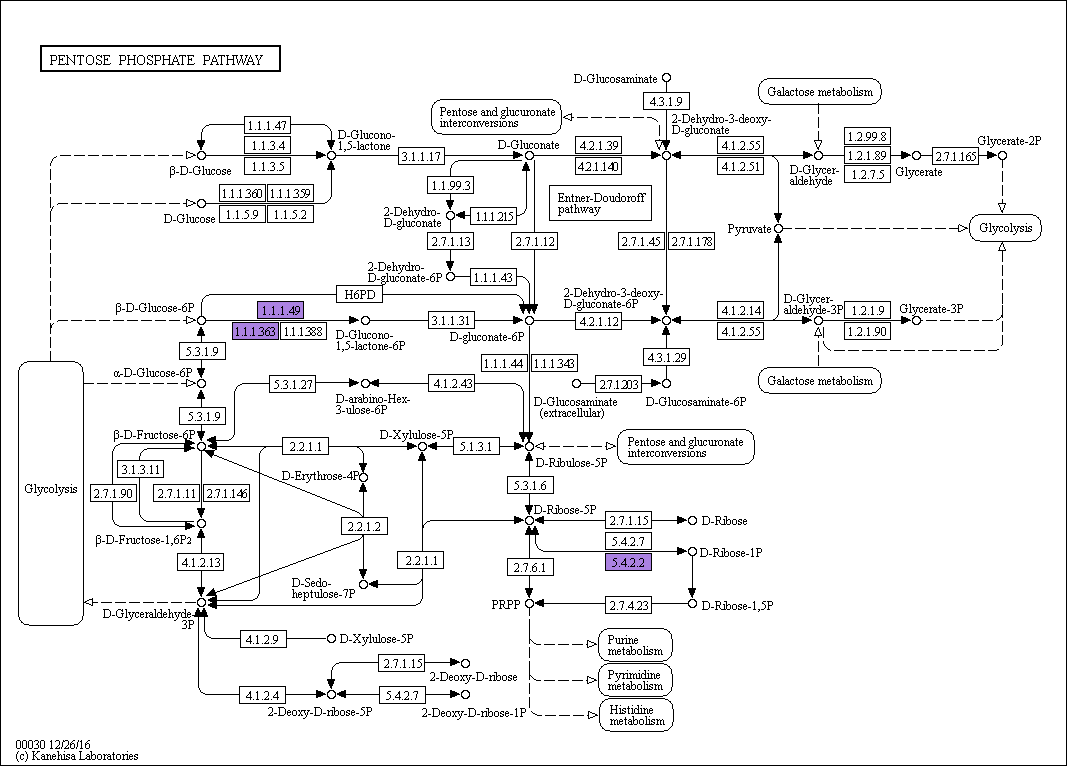


**Supplementary Figure 7** Four genes encoding phosphoproteins represent three enzymes of the pentose phosphate pathway; and are E1.1.1.49 (Glyma.16g063200), E1.1.1.363 (Glyma.19g082300), E5.4.2.2 (Glyma.08g044100 and Glyma.05g237000).


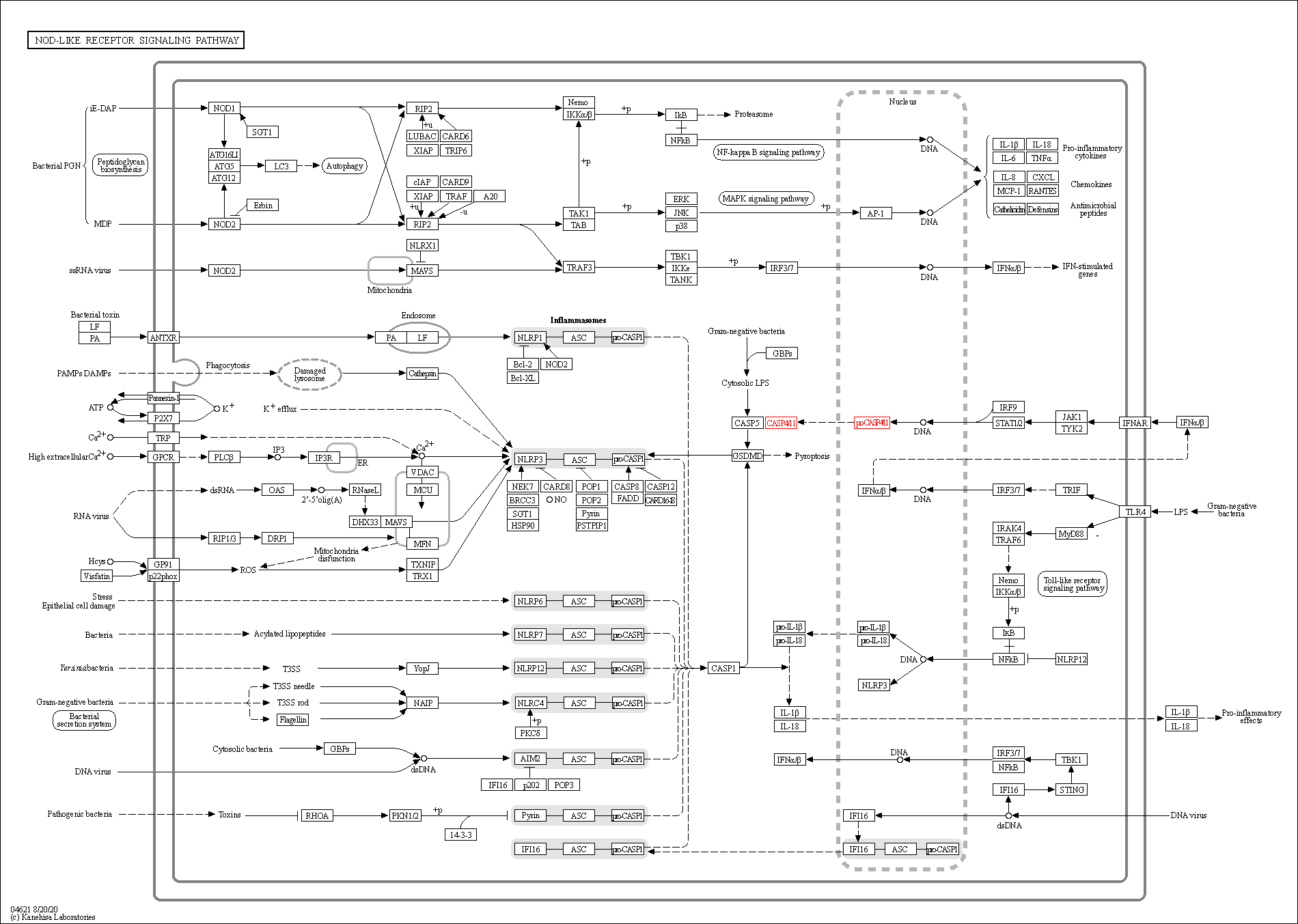


**Supplementary Figure 8** Two type II metacaspases involved in cell death pathway. Glyma.08G233300 and Glyma.08G233500 are two type II metacaspases, highly similar to caspase 4.

**Supplementary Figure 9** The phosphopeptides of the type II metacaspases that showed reduced phosphorylation levels in the *rn1* mutant. Type II metacaspases, Glyma.08G233300.1 and Glyma.08G233500.1 (Table 2), and Glyma.15G219100.1 (Supplemental Table S2) showed reduced phosphorylation in the serine residues (red font) of the underlined phosphopeptides located in the P20 caspase-like domain presented with black font. The linker domain is shown with blue font and P10 caspase-like domain with grey font. C-terminal region of Glyma.08G233300.1 was shown to interact with the N-terminal region of the *Phytophthora* resistance protein Rps1-k-2 (Gao 2006; Baskett 2012).

**Supplementary Figure 10** GO and KEGG analysis of 24 phosphopeptides from less abundant phosphoproteins. (A) The 24 phosphopeptides presented in Table 3 were classified into three categories: biological process, cellular component, and molecular function, based on GO terms. (B) Specific pathways and number of phosphopeptides in each pathway classified under each of the three categories. One phosphopeptide is significantly involved in immune system process and plant hormone signal transduction, indicated by “*”.

**Supplementary Figure 11** MapMan analysis of 24 phosphopeptides from less abundant phosphoproteins. Four phosphopeptides induced by biotic and abiotic stresses are shown on both sides of the map.
