# Supplementary material for: A Phosphoproteomics Study of the Soybean root necrosis 1 Mutant Revealed Type II Metacaspases Involved in Cell Death Pathway
Source: Front Plant Sci. 2022 Jul 19;13:882561. doi: 10.3389/fpls.2022.882561 (PMC9344878; doi:10.3389/fpls.2022.882561)
Supplement: Supplementary file 2 [file Data_Sheet_2.docx]

**
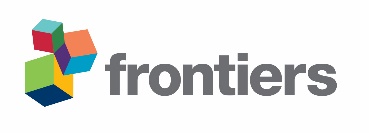
**

Supplementary Material

## Supplementary Tables

| **Supplementary Table 1** The identified 146 phosphorylated proteins. | | | | | |
| --- | --- | --- | --- | --- | --- |
| Gene ID | ^1^Fold Change | ^2^Transcript Number | Homologue | Annotation |  |
| Glyma.01G015500.1 | 1.05 ± 0.33 | 33.46 | AT4G17720.1 | RNA-binding (RRM/RBD/RNP motifs) family protein |  |
| Glyma.01G022900.1 | 0.88 ± 0.19 | 14.52 | AT2G22560.1 | Kinase interacting (KIP1-like) family protein |  |
| Glyma.01G036700.1 | 0.84 ± 0.20 | 16.02 | AT1G70180.2 | Sterile alpha motif (SAM) domain-containing protein |  |
| Glyma.01G057100.1 | 0.86 ± 0.19 | 170.56 | AT3G29360.1 | UDP-glucose 6-dehydrogenase family protein |  |
| Glyma.01G119500.1 | 0.83 ± 0.28 | 2.37 | AT2G38280.1 | AMP deaminase, putative / myoadenylate deaminase |  |
| Glyma.01G136000.1 | 1.04 ± 0.37 | 43.69 | AT2G45140.1 | Plant VAP homolog 12 |  |
| Glyma.01G144400.1 | 1.00 ± 0.27 | 11.23 | AT2G45430.1 | AT-hook motif nuclear-localized protein 22 |  |
| Glyma.01G161100.1 | 1.25 ± 0.39 | 99.59 | AT1G47670.1 | Transmembrane amino acid transporter family protein |  |
| Glyma.01G162000.1 | 1.07 ± 0.29 | 42.85 | AT4G35785.2 | RNA-binding (RRM/RBD/RNP motifs) family protein |  |
| Glyma.01G175100.1 | 0.96 ± 0.21 | 17.48 | AT3G50670.1 | U1 small nuclear ribonucleoprotein-70K |  |
| Glyma.01G207400.1 | 1.10 ± 0.31 | 3.52 | AT2G39810.1 | Ubiquitin-protein ligases |  |
| Glyma.01G213100.1 | 1.08 ± 0.43 | 14.24 | AT2G45430.1 | AT-hook motif nuclear-localized protein 22 |  |
| Glyma.01G230700.1 | 0.99 ± 0.31 | 5.12 | AT1G33680.1 | KH domain-containing protein |  |
| Glyma.01G232500.1 | 1.21 ± 0.36 | 35.4 | AT3G03050.1 | Cellulose synthase-like D3 |  |
| Glyma.02G021600.1 | 0.78 ± 0.19 | 197.7 | AT1G07040.1 | Unknown function |  |
| Glyma.02G073600.1 | 0.89 ± 0.33 | 25.18 | AT2G37170.1 | Plasma membrane intrinsic protein 2 |  |
| Glyma.02G216000.1 | 0.87 ± 0.33 | 3.71 | AT1G55860.2 | Ubiquitin-protein ligase 1 |  |
| Glyma.02G240400.1 | 0.71 ± 0.19 | 0.77 | AT1G73370.1 | Sucrose synthase 6 |  |
| Glyma.02G291600.1 | 1.09 ± 0.32 | 5.89 | AT1G28490.1 | Syntaxin of plants 61 |  |
| Glyma.02G311300.1 | 1.03 ± 0.05 | 11.56 | AT1G21630.1 | Calcium-binding EF hand family protein |  |
| Glyma.03G002400.1 | 0.81 ± 0.21 | 29.66 | AT5G27640.1 | Translation initiation factor 3B1 |  |
| Glyma.03G017200.1 | 0.88 ± 0.15 | 11.71 | AT5G52040.2 | RNA-binding (RRM/RBD/RNP motifs) family protein |  |
| Glyma.03G070400.1 | 0.84 ± 0.15 | 15.31 | AT3G09350.1 | Fes1A |  |
| Glyma.03G076500.1 | 0.86 ± 0.25 | 12.73 | AT3G54130.1 | Josephin family protein |  |
| Glyma.03G089600.1 | 1.03 ± 0.09 | 8.06 | AT3G07440.1 | Unknown function |  |
| Glyma.03G163600.1 | 0.66 ± 0.22 | 35.18 | AT1G06210.1 | ENTH/VHS/GAT family protein |  |
| Glyma.03G167100.1 | 0.79 ± 0.29 | 12.26 | AT5G43960.1 | Nuclear transport factor 2 (NTF2) family protein with RNA binding (RRM-RBD-RNP motifs) domain |  |
| Glyma.03G239900.1 | 1.16 ± 0.46 | 10.97 | AT3G05200.1 | RING/U-box superfamily protein |  |
| Glyma.03G252300.1 | 0.94 ± 0.23 | 2.09 | AT1G64790.1 | ILITYHIA |  |
| Glyma.04G016600.1 | 1.09 ± 0.20 | 20.01 | AT1G20760.1 | Calcium-binding EF hand family protein |  |
| Glyma.04G038600.1 | 0.95 ± 0.25 | 6.29 | AT2G26570.1 | Plant protein of unknown function (DUF827) |  |
| Glyma.04G066800.1 | 0.89 ± 0.25 | 8.38 | AT4G32330.2 | TPX2 (targeting protein for Xklp2) protein family |  |
| Glyma.04G070200.1 | 0.94 ± 0.09 | 3.05 | AT4G32551.2 | Lish dimerisation motif; WD40/YVTN repeat-like |  |
| Glyma.04G083900.1 | 0.95 ± 0.21 | 6.63 | AT5G11600.1 | Unknown function |  |
| Glyma.04G171300.1 | 0.92 ± 0.22 | 12.52 | AT5G62500.1 | End binding protein 1B |  |
| Glyma.04G200100.1 | 0.81 ± 0.30 | 8.48 | AT3G48860.2 | Unknown function |  |
| Glyma.04G243600.1 | 1.19 ± 0.16 | 77.43 | AT1G21380.1 | Target of Myb protein 1 |  |
| Glyma.05G003400.1 | 1.04 ± 0.39 | 11.94 | AT5G40490.1 | RNA-binding (RRM/RBD/RNP motifs) family protein |  |
| Glyma.05G005500.1 | 0.79 ± 0.21 | 6.72 | AT5G14610.2 | DEAD box RNA helicase family protein |  |
| Glyma.05G011600.1 | 0.75 ± 0.27 | 3.64 | AT2G26730.1 | Leucine-rich repeat protein kinase family protein |  |
| Glyma.05G026600.1 | 1.1 ± 0.30 | 6.28 | AT5G62440.1 | Protein of unknown function (DUF3223) |  |
| Glyma.05G070300.1 | 1.02 ± 0.41 | 181.35 | AT1G32400.2 | Tobamovirus multiplication 2A |  |
| Glyma.05G131100.1 | 1.01 ± 0.33 | 5.23 | AT5G60620.1 | Glycerol-3-phosphate acyltransferase 9 |  |
| Glyma.05G139400.1 | 0.95 ± 0.23 | 5.1 | AT3G18240.1 | Ribosomal protein S24/S35, mitochondrial |  |
| Glyma.05G174300.1 | 0.90 ± 0.17 | 135.83 | AT5G57290.1 | 60S acidic ribosomal protein family |  |
| Glyma.05G231500.1 | 1.32 ± 0.47 | 3.77 | AT1G67910.1 | Unknown function |  |
| Glyma.05G237000.1 | 0.66 ± 0.14 | 73.37 | AT1G70730.1 | Phosphoglucomutase/phosphomannomutase family protein |  |
| Glyma.05G248500.1 | 0.80 ± 0.43 | 3.63 | AT1G73460.1 | Protein kinase superfamily protein |  |
| Glyma.06G023000.1 | 1.13 ± 0.36 | 20.49 | AT1G20110.1 | RING/FYVE/PHD zinc finger superfamily protein |  |
| Glyma.06G163900.1 | 0.99 ± 0.28 | 8.24 | AT5G50970.1 | Transducin family protein / WD-40 repeat family protein |  |
| Glyma.06G243800.1 | 1.09 ± 0.36 | 0 | AT1G07650.2 | Leucine-rich repeat transmembrane protein kinase |  |
| Glyma.06G255200.1 | 0.90 ± 0.20 | 54.96 | AT1G11360.4 | Adenine nucleotide alpha hydrolases-like superfamily protein |  |
| Glyma.06G311400.1 | 1.08 ± 0.29 | 10.09 | AT3G12480.1 | Nuclear factor Y, subunit C11 |  |
| Glyma.07G017700.1 | 0.75 ± 0.40 | 17.08 | AT3G16270.1 | ENTH/VHS family protein |  |
| Glyma.07G023400.1 | 0.73 ± 0.14 | 40.76 | AT3G58730.1 | Vacuolar ATP synthase subunit D (VATD) / V-atpase, D subunit / vacuolar proton pump D subunit (VATPD) |  |
| Glyma.07G056700.1 | 0.88 ± 0.29 | 4.38 | AT3G61690.1 | Nucleotidyltransferases |  |
| Glyma.07G070900.1 | 0.83 ± 0.22 | 14.08 | AT1G31870.1 | Unknown function |  |
| Glyma.07G144300.1 | 1.01 ± 0.32 | 8.68 | AT5G01160.1 | RING/U-box superfamily protein |  |
| Glyma.07G159600.1 | 0.80 ± 0.24 | 21.39 | AT1G20970.1 | Unknown function |  |
| Glyma.07G237900.1 | 1.28 ± 0.35 | 7.85 | AT4G16280.2 | RNA binding;abscisic acid binding |  |
| Glyma.07G273100.1 | 1.01 ± 0.35 | 5.58 | AT1G59610.1 | Dynamin-like 3 |  |
| Glyma.08G005900.1 | 0.99 ± 0.34 | 10.1 | AT5G51060.1 | NADPH/respiratory burst oxidase protein D |  |
| Glyma.08G026700.1 | 1.30 ± 0.64 | 10.79 | AT2G07360.1 | SH3 domain-containing protein |  |
| Glyma.08G044100.1 | 0.63 ± 0.18 | 43.56 | AT1G70730.1 | Phosphoglucomutase/phosphomannomutase family protein |  |
| Glyma.08G109600.1 | 1.09 ± 0.35 | 6.13 | AT3G23900.1 | RNA recognition motif (RRM)-containing protein |  |
| Glyma.08G233300.1 | 0.85 ± 0.24 | 68.22 | AT1G79330.1 | Metacaspase 5 |  |
| Glyma.08G233500.1 | 0.83 ± 0.24 | 73.95 | AT1G79340.1 | Metacaspase 4 |  |
| Glyma.08G243000.1 | 0.83 ± 0.19 | 149.5 | AT5G15490.1 | UDP-glucose 6-dehydrogenase family protein |  |
| Glyma.08G293900.1 | 1.43 ± 0.40 | 10.33 | AT1G59820.1 | Aminophospholipid atpase 3 |  |
| Glyma.08G297500.1 | 0.70 ± 0.18 | 2.74 | AT1G14380.1 | IQ-domain 28 |  |
| Glyma.08G330800.1 | 0.88 ± 0.24 | 16.24 | AT5G55860.1 | Plant protein of unknown function (DUF827) |  |
| Glyma.08G362400.1 | 1.20 ± 0.52 | 49.03 | AT2G43160.1 | ENTH/VHS family protein |  |
| Glyma.09G002100.1 | 1.09 ± 0.33 | 15.83 | AT1G59610.1 | Dynamin-like 3 |  |
| Glyma.09G056300.1 | 0.96 ± 0.37 | 214.38 | AT4G30190.1 | H(+)-atpase 2 |  |
| Glyma.09G057400.1 | 0.90 ± 0.22 | 31.56 | AT4G30160.1 | Villin 4 |  |
| Glyma.09G101200.1 | 1.01 ± 0.20 | 5.22 | AT3G20550.1 | SMAD/FHA domain-containing protein |  |
| Glyma.09G103000.1 | 0.78 ± 0.42 | 75.63 | AT5G05170.1 | Cellulose synthase family protein |  |
| Glyma.09G142100.1 | 1.06 ± 0.17 | 26.71 | AT5G52200.1 | Phosphoprotein phosphatase inhibitors |  |
| Glyma.09G174000.1 | 1.15 ± 0.36 | 15.86 | AT3G17850.1 | Protein kinase superfamily protein |  |
| Glyma.09G204100.1 | 0.76 ± 0.11 | 18.07 | AT4G17870.1 | Polyketide cyclase/dehydrase and lipid transport superfamily protein |  |
| Glyma.09G235900.1 | 0.98 ± 0.33 | 12.77 | AT5G15270.2 | RNA-binding KH domain-containing protein |  |
| Glyma.09G265000.1 | 0.90 ± 0.33 | 3.58 | AT4G00740.1 | S-adenosyl-L-methionine-dependent methyltransferases superfamily protein |  |
| Glyma.09G273200.1 | 0.91 ± 0.32 | 145.48 | AT3G08710.1 | Thioredoxin H-type 9 |  |
| Glyma.10G034800.1 | 0.74 ± 0.24 | 16.16 | AT3G58170.1 | BET1P/SFT1P-like protein 14A |  |
| Glyma.10G100400.1 | 1.01 ± 0.15 | 7.65 | AT2G41900.1 | CCCH-type zinc finger protein with ARM repeat domain |  |
| Glyma.10G140000.1 | 0.93 ± 0.09 | 11.11 | AT1G02330.1 | Unknown function |  |
| Glyma.10G167100.1 | 1.18 ± 0.29 | 18.25 | AT4G14465.1 | AT-hook motif nuclear-localized protein 20 |  |
| Glyma.10G173000.1 | 0.93 ± 0.21 | 21.61 | AT4G24100.1 | Protein kinase superfamily protein |  |
| Glyma.10G208800.1 | 0.93 ± 0.44 | 15.33 | AT2G39130.1 | Transmembrane amino acid transporter family protein |  |
| Glyma.10G210700.1 | 0.89 ± 0.11 | 5.12 | AT5G17530.3 | Phosphoglucosamine mutase family protein |  |
| Glyma.10G214700.1 | 1.20 ± 0.45 | 75.14 | AT4G09510.1 | Cytosolic invertase 2 |  |
| Glyma.10G239100.1 | 0.70 ± 0.21 | 21.36 | AT4G25500.1 | Arginine/serine-rich splicing factor 35 |  |
| Glyma.10G247200.1 | 0.86 ± 0.17 | 13.97 | AT3G22540.1 | Protein of unknown function (DUF1677) |  |
| Glyma.10G255700.1 | 0.62 ± 0.23 | 3.11 | AT3G19670.1 | Pre-mrna-processing protein 40B |  |
| Glyma.10G257200.1 | 2.11 ± 1.06 | 59.63 | AT3G19615.1 | Unknown function |  |
| Glyma.10G263700.1 | 0.96 ± 0.22 | 36.21 | AT3G19420.1 | PTEN 2 |  |
| Glyma.11G078100.1 | 0.89 ± 0.28 | 44.7 | AT4G38630.1 | Regulatory particle non-atpase 10 |  |
| Glyma.11G114300.1 | 0.96 ± 0.26 | 31.82 | AT3G05060.1 | NOP56-like pre RNA processing ribonucleoprotein |  |
| Glyma.11G139600.1 | 0.84 ± 0.24 | 40.09 | AT3G46540.1 | ENTH/VHS family protein |  |
| Glyma.11G237500.1 | 0.92 ± 0.27 | 16.47 | AT4G39680.2 | SAP domain-containing protein |  |
| Glyma.12G001300.1 | 0.98 ± 0.25 | 1.8 | AT1G57820.1 | Zinc finger (C3HC4-type RING finger) family protein |  |
| Glyma.12G095300.1 | 0.82 ± 0.28 | 4.43 | AT1G08420.1 | BRI1 suppressor 1 (BSU1)-like 2 |  |
| Glyma.12G146100.1 | 1.04 ± 0.31 | 19.23 | AT1G11360.2 | Adenine nucleotide alpha hydrolases-like superfamily protein |  |
| Glyma.12G226300.1 | 0.87 ± 0.26 | 5.95 | AT3G15220.1 | Protein kinase superfamily protein |  |
| Glyma.12G242300.1 | 0.90 ± 0.25 | 17.89 | AT1G59610.1 | Dynamin-like 3 |  |
| Glyma.13G057700.1 | 0.78 ± 0.08 | 133.97 | AT3G29360.1 | UDP-glucose 6-dehydrogenase family protein |  |
| Glyma.13G346900.1 | 1.23 ± 0.42 | 13.43 | AT3G14010.3 | CTC-interacting domain 4 |  |
| Glyma.13G357600.1 | 0.78 ± 0.32 | 0.52 | AT1G45688.1 | Unknown function |  |
| Glyma.13G361900.1 | 1.13 ± 0.11 | 36.21 | AT1G15520.1 | Pleiotropic drug resistance 12 |  |
| Glyma.14G146200.1 | 0.84 ± 0.17 | 7.58 | AT5G11600.1 | Unknown function |  |
| Glyma.15G011900.1 | 1.18 ± 0.35 | 75.6 | AT1G15520.1 | Pleiotropic drug resistance 12 |  |
| Glyma.15G056100.1 | 0.70 ± 0.22 | 11.02 | AT3G57150.1 | Homologue of NAP57 |  |
| Glyma.15G077600.1 | 1.07 ± 0.76 | 42.52 | AT2G33850.1 | Unknown function |  |
| Glyma.15G163700.1 | 0.77 ± 0.09 | 29.02 | AT4G30160.1 | Villin 4 |  |
| Glyma.15G219100.1 | 0.69 ± 0.21 | 89.49 | AT1G79340.1 | Metacaspase 4 |  |
| Glyma.15G227300.1 | 1.15 ± 0.28 | 16.31 | AT3G12480.1 | Nuclear factor Y, subunit C11 |  |
| Glyma.15G261200.1 | 0.88 ± 0.35 | 36.59 | AT1G16270.2 | Protein kinase superfamily protein with octicosapeptide /Phox/Bem1p domain |  |
| Glyma.15G272900.1 | 1.02 ± 0.35 | 6.72 | AT3G12640.1 | RNA binding (RRM/RBD/RNP motifs) family protein |  |
| Glyma.16G037900.1 | 0.58 ± 0.20 | 731.66 | - | Unknown function |  |
| Glyma.16G063200.1 | 1.00 ± 0.25 | 151.77 | AT5G40760.1 | Glucose-6-phosphate dehydrogenase 6 |  |
| Glyma.16G212900.1 | 1.28 ± 0.29 | 98.69 | AT1G65730.1 | YELLOW STRIPE like 7 |  |
| Glyma.17G075200.1 | 1.06 ± 0.31 | 48.49 | AT2G24260.1 | LJRHL1-like 1 |  |
| Glyma.17G098100.1 | 1.00 ± 0.27 | 4.05 | AT5G62550.1 | Unknown function |  |
| Glyma.17G112500.1 | 0.98 ± 0.52 | 12.18 | AT3G15880.2 | WUS-interacting protein 2 |  |
| Glyma.17G131100.1 | 0.98 ± 0.18 | 17.1 | AT4G11740.1 | Ubiquitin-like superfamily protein |  |
| Glyma.17G136600.1 | 1.07 ± 0.27 | 6.98 | AT4G22810.1 | Predicted AT-hook DNA-binding family protein |  |
| Glyma.17G218800.1 | 0.88 ± 0.16 | 7.68 | AT4G33060.1 | Cyclophilin-like peptidyl-prolyl cis-trans isomerase family protein |  |
| Glyma.17G249900.1 | 0.95 ± 0.28 | 2.48 | AT5G42950.1 | GYF domain-containing protein |  |
| Glyma.17G254200.1 | 1.25 ± 0.40 | 301.45 | AT3G51030.1 | Thioredoxin H-type 1 |  |
| Glyma.18G055200.1 | 1.10 ± 0.44 | 16.05 | AT2G23140.1 | RING/U-box superfamily protein with ARM repeat domain |  |
| Glyma.18G102900.1 | 1.06 ± 0.22 | 3.46 | AT3G20550.1 | SMAD/FHA domain-containing protein |  |
| Glyma.18G195500.1 | 0.86 ± 0.18 | 7.11 | AT5G01160.1 | RING/U-box superfamily protein |  |
| Glyma.18G219100.1 | 0.90 ± 0.16 | 88.83 | AT3G08590.1 | Phosphoglycerate mutase, 2,3-bisphosphoglycerate-independent |  |
| Glyma.18G225900.1 | 0.77 ± 0.25 | 3.72 | AT4G00740.1 | S-adenosyl-L-methionine-dependent methyltransferases superfamily protein |  |
| Glyma.18G242700.1 | 0.79 ± 0.24 | 16.21 | AT3G02750.1 | Protein phosphatase 2C family protein |  |
| Glyma.18G299500.1 | 1.15 ± 0.42 | 19.93 | AT2G43160.1 | ENTH/VHS family protein |  |
| Glyma.19G006000.1 | 0.80 ± 0.21 | 13.65 | AT5G14610.2 | DEAD box RNA helicase family protein |  |
| Glyma.19G009300.1 | 1.08 ± 0.26 | 16.61 | AT5G27990.1 | Pre-rrna-processing protein TSR2, conserved region |  |
| Glyma.19G012300.1 | 0.83 ± 0.26 | 20.08 | AT3G48050.2 | BAH domain;TFIIS helical bundle-like domain |  |
| Glyma.19G025400.1 | 1.19 ± 0.66 | 85.25 | AT5G39570.1 | Unknown function |  |
| Glyma.19G082300.1 | 1.00 ± 0.34 | 81.07 | AT5G40760.1 | Glucose-6-phosphate dehydrogenase 6 |  |
| Glyma.19G168200.1 | 0.91 ± 0.37 | 17.56 | AT5G43960.1 | Nuclear transport factor 2 (NTF2) family protein with RNA binding (RRM-RBD-RNP motifs) domain |  |
| Glyma.20G049400.1 | 0.92 ± 0.19 | 4.89 | AT4G12640.1 | RNA recognition motif (RRM)-containing protein |  |
| Glyma.20G116300.1 | 0.93 ± 0.27 | 15.57 | AT1G25420.1 | Regulator of Vps4 activity in the MVB pathway protein |  |
| Glyma.20G153600.1 | 0.84 ± 0.23 | 3.23 | AT5G51820.1 | Phosphoglucomutase |  |
| Glyma.20G223500.1 | 0.87 ± 0.29 | 7.15 | AT3G04470.1 | Ankyrin repeat family protein |  |

^1^Fold Change was referred the ratio of the phosphorylated proteins expression between necrotic and healthy roots (Necrotic/Healthy)

^2^Transcript Number: The FPKM value of the genes encoded phosphorylated proteins in root tissue of *G. m*ax. Williams82 (Severin et al., 2010).

**Supplementary Table 2** Differential phosphorylation of phosphopetides of detectible phosphoproteins.
